# Fish Consumption and Colorectal Cancer Risk: Meta-Analysis of Prospective Epidemiological Studies and Review of Evidence from Animal Studies

**DOI:** 10.3390/cancers14030640

**Published:** 2022-01-27

**Authors:** Saverio Caini, Sofia Chioccioli, Elisa Pastore, Miriam Fontana, Katia Tortora, Giovanna Caderni, Giovanna Masala

**Affiliations:** 1 Institute for Cancer Research, Prevention and Clinical Network (ISPRO), Via Cosimo il Vecchio 2, 50139 Florence, Italy; s.caini@ispro.toscana.it (S.C.); m.fontana@ispro.toscana.it (M.F.); k.tortora@ispro.toscana.it (K.T.); g.masala@ispro.toscana.it (G.M.); 2 NEUROFARBA Department, Pharmacology and Toxicology Section, University of Florence, Viale Pieraccini 6, 50139 Florence, Italy; sofia.chioccioli@unifi.it (S.C.); giovanna.caderni@unifi.it (G.C.)

**Keywords:** fish consumption, colorectal cancer, epidemiological studies, animal studies, review, meta-analysis

## Abstract

**Simple Summary:**

We meta-analyzed prospective epidemiological studies reporting on the association between fish consumption and colorectal cancer (CRC) risk among humans and reviewed preclinical studies that examined the link between fish components and colorectal carcinogenesis in animals. By pooling results from 25 studies (encompassing over 25,000 CRC cases) published up to November 2020, we found convincing evidence that increased fish consumption may protect from CRC development among humans. The review of animal studies allowed identifying several biological mechanisms able to explain the associations that have emerged in human populations. Dietary recommendations for cancer prevention should incorporate the evidence from this literature review and meta-analysis.

**Abstract:**

**Background**: Epidemiological studies on the association between fish consumption and colorectal cancer (CRC) risk have yielded inconsistent results, despite evidence from preclinical studies that long-chain ω-3 polyunsaturated fatty acids inhibit colorectal carcinogenesis. We conducted a meta-analysis of prospective epidemiological studies investigating the association between fish consumption and CRC risk among humans and reviewed studies examining the link between fish components and colorectal carcinogenesis in animal models. **Methods**: We included studies published until November 2020. We calculated the summary risk ratio (SRR) and 95% confidence intervals (CI) through random effects meta-analysis models in order to summarize evidence from studies among humans. **Results**: Twenty-five prospective epidemiological studies encompassing 25,777 CRC cases were included. Individuals in the highest (vs. lowest) category of fish consumption had a significantly reduced risk of CRC (SRR 0.94, 95%CI 0.89–0.99). In dose–response meta-analysis, a 50-g increment in the daily consumption of fish was associated with a statistically significant 4% reduction in CRC risk (SRR 0.96, 95%CI 0.92–0.99). Preclinical studies (n = 25) identified multiple mechanisms of action of fish and fish components on colorectal carcinogenesis. **Conclusions**: Dietary recommendations for cancer prevention should take into account the evidence from epidemiological and preclinical studies that increasing fish consumption may be effective in preventing CRC.

## 1. Introduction

Colorectal cancer (CRC) is one of the most common and deadliest malignancies worldwide. According to the Global Cancer Observatory (GCO) data for 2020, it ranks third globally in terms of incidence, with an estimated 1,931,590 new cases, and second in terms of mortality, causing 935,173 deaths [1]. CRC disease burden is generally higher in high-income industrialized countries [1], clearly suggesting the importance of lifestyle factors in its etiology. 

The role of diet in the pathogenesis of CRC is now established, yet uncertainty remains about some specific foods and food classes. In the latest (2018) report of the World Cancer Research Fund (WCRF) on diet, nutrition, physical activity, and CRC [2], the Expert Panel concluded there was strong evidence that red and processed meat, alcoholic drinks, and body fatness increase CRC risk, while whole grains, foods containing dietary fiber, and dairy products have a protective effect. The evidence suggesting that fish consumption reduces CRC risk was judged as still limited, albeit generally consistent. 

Fish is a key component of the Mediterranean diet, and its consumption is usually actively encouraged as it contains many high-quality proteins and essential micronutrients (vitamins and minerals) and fatty acids [3]. In particular, fish represents the main dietary source of long-chain ω-3 polyunsaturated fatty acids (ω-3 PUFA), among which are eicosapentaenoic acid (EPA) and docosahexaenoic acid (DHA), which possess anti-inflammatory and immunomodulatory properties [4]. Studies in animal models have consistently suggested that fish oil (FO) containing ω-3 PUFA inhibits colorectal carcinogenesis through several pathways [5,6], and a recently published meta-analysis confirmed these findings among humans by highlighting an inverse relationship between ω-3 PUFA intake, blood levels, and CRC risk [7]. Epidemiological studies on the association between fish consumption and CRC risk in human populations did not always yield consistent results [8,9,10,11], and previous meta-analyses aggregating the available evidence from prospective investigations failed to reach conclusive results, highlighting the need for further research [12].

Recently, findings from large population-based cohort studies have been published [13,14]; hence, conducting an updated meta-analysis seems appropriate and timely. Moreover, we believe that coupling the evidence from epidemiological studies with that from preclinical studies may greatly help to jointly evaluate the robustness and plausibility of the hypothesis under study. Therefore, we conducted an up-to-date meta-analysis of prospective studies on the association between fish consumption and CRC risk among humans and a review of the available evidence on the link between the intake of fish and fish components and colorectal carcinogenesis in animal models.

## 2. Materials and Methods

### 2.1. Fish Consumption and Colorectal Cancer Risk among Humans

#### 2.1.1. Literature Search and Inclusion Criteria

The literature search and statistical analysis were conducted according to the MOOSE guidelines for the meta-analyses of observational studies [15] and the protocol was registered in the Prospective Register of Systematic Reviews (PROSPERO CRD42022299629). On 23 November 2020, we searched PUBMED/MEDLINE and EMBASE using the following search string: (fish OR seafood* OR shellfish) AND (colon OR rectal OR rectum OR colorectal) AND (cancer OR carcinoma OR tumour OR tumor OR malignancy). After removing duplicates, papers were initially screened based on their title and abstract; those that were considered as potentially of interest for the present meta-analysis were then obtained and read in full text to decide on their eligibility. Additional papers were searched by the backward citation chaining of eligible papers and previously published literature reviews and meta-analysis. Papers were eligible if they had a prospective study design (cohort, nested case-control studies (NCC), and randomized clinical trials (RCT)) and reported a measure of relative risk (RR) (e.g., hazard ratio or odds ratio), a corresponding 95% confidence intervals (CI), or a different measure of statistical uncertainty (e.g., standard errors, variance, or exact *p*-value) for the association between the consumption of fish and the risk of colon, rectal, or colorectal cancer. Retrospective case–control studies were not considered, as well as ecological studies, case reports, editorials/letters that did not present original data, and studies with any design in which the outcome of interest was cancer mortality instead of cancer risk. In the case of non-independent studies (with total overlap, e.g., two or more reports from the same cohort, or with partial overlap, e.g., one study reporting from a cohort consortium and a different study presenting results for a cohort that is part of it), the older/smaller study was discarded unless it provided RR estimates that were not available in a more recent/larger study. Two researchers independently decided on the inclusion of each paper and any disagreement was resolved by consensus. 

As illustrated in more detail below (see Results), the type of food studied in relation to CRC risk varied across articles in that it could be limited to fish (sometimes left unspecified) or encompass a varying combination of fish and other seafoods including crustaceans and mollusks/shellfish. For the sake of brevity, the generic term “fish” will be used henceforth to refer to the area of interest.

#### 2.1.2. Data Extraction and Statistical Analysis

The following information was extracted from all eligible papers: first author and year of publication; country in which the study was conducted; study design; source; sex and age distribution of the whole study population and number of cancer cases (for cohort studies and RCT), or of cancer cases and controls (for NCC); details of matching, if any, for NCC; methods for data collection on food consumption; mean/median and maximum duration of follow-up and total number of person–years accrued; distribution of cancer cases by site (colon further split into proximal and distal colon, rectum, or other, and overlapping sites); adjusted (whenever available) or unadjusted RRs and their 95% CI, alongside the number of cases and non-cases/controls and the total number of person–years in each category of exposure (e.g., each quantile of fish consumption); details on the statistical methods and the variables used for adjustment.

RR estimates and corresponding 95% confidence intervals were transformed into log relative risk (logRR) and corresponding variance as proposed by Greenland [16]. We then conducted highest versus lowest and linear and non-linear dose–response random effects meta-analyses to summarize the evidence on the association between fish consumption and CRC risk. For linear dose–response meta-analysis, we followed the method described by Greenland and Longnecker [17] to compute a trend from RRs and 95% CI calculated across categories (e.g., quantiles) of exposure, when this was not directly provided in the paper. The non-linear dose–response meta-analysis was conducted using 3-knots restricted cubic splines (5th, 50th, and 95th percentiles of the distribution). Between studies, the heterogeneity of RR estimates was quantified using the I^2^ statistics, which is a measure of the total variation of effects across studies that can be attributed to actual heterogeneity rather than chance [18]. When I^2^ was above 50% (which denotes large heterogeneity) and the sample size allowed, we conducted subgroup analyses and fitted meta-regression models (for categorical and continuous variables, respectively) to determine what study characteristics could explain a significant share of the observed heterogeneity. Analyses stratified by sex, cancer site (colon vs. rectum), and country were, however, planned a priori and conducted regardless of whether or not there was substantial heterogeneity. Publication bias was assessed by visually inspecting the funnel plot and applying the Egger’s test [19]. Finally, the methodological quality and proneness to bias of all included studies was rated using the Newcastle–Ottawa Scale (NOS) [20].

Statistical analyses were conducted using Stata (version 14, command metan, for highest versus lowest meta-analysis) and R (version 4.0.0, command dosresmeta [21] for dose–response meta-analysis) software.

### 2.2. Fish and Fish-Oil Intake and Colorectal Carcinogenesis in Animal Models 

The literature search for preclinical studies focusing on the relationship between the intake of fish, FO intake, and colon carcinogenesis in animal models was conducted in PUBMED/MEDLINE using the following search string: (chemoprevention OR omega3 OR “omega 3” OR omega-3 OR diet OR fish) AND (OR colon OR colorectal OR crc OR intestinal OR bowel) AND (cancer* OR carcinogenesis) AND (rodent* OR rat* OR mouse OR mice). The search was conducted on 3 May 2021, and it extends, therefore, to all the articles that were published until April 2021. To be included in the final review, an article needed to report original data and examine the effect of fish and/or fish oil intake in the process of colorectal carcinogenesis and metastasis in rodent models. The article selection was conducted according to the same procedures described above; no formal meta-analysis was conducted because of the great diversity across studies in terms of several characteristics (see Section 3). 

## 3. Results

### 3.1. Fish Consumption and Colorectal Cancer Risk among Humans

**Figure 1:**
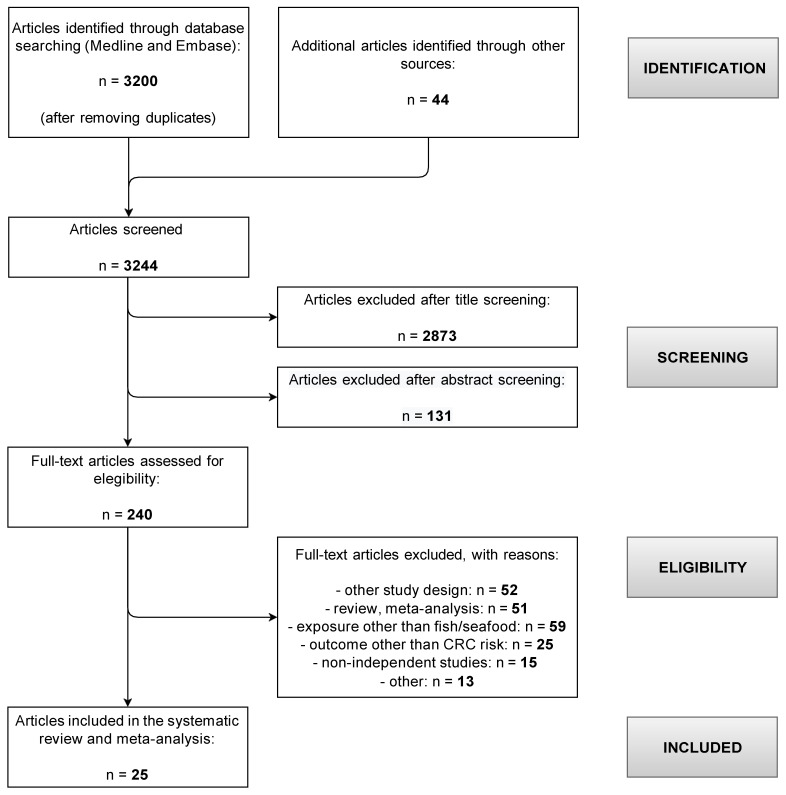
Flow-chart for the selection of articles included in the systematic review and meta-analysis on the association between fish consumption and colorectal cancer risk.

The literature search returned 3200 non-duplicate entries, and 44 were added via backward reference searching (Figure 1).

A total of 2873 and 131 papers were screened out based on their titles and abstract, and the remaining 240 papers were read in full copy. Finally, 25 articles reporting on the association between fish consumption and CRC risk met the inclusion criteria for the present meta-analysis [8,9,10,11,13,14,22,23,24,25,26,27,28,29,30,31,32,33,34,35,36,37,38,39,40] The papers by Bamia et al. [22] and Engeset et al. [23] were totally and partially overlapping, respectively, with the larger and more recent paper by Aglago et al. [13], which reported from the large, multi-country European Prospective Investigation into Cancer and Nutrition (EPIC) cohort study. Likewise, the two papers by Murff et al. [24] and Lee et al. [11] were based on the same prospective cohort (Shanghai Women’s Health Study). The study by Spencer et al. [25], a case–control study nested within a consortium of UK-based dietary cohorts including the EPIC-Norfolk and the EPIC-Oxford cohorts, had only a limited overlap with that by Aglago et al. [13], to which the two English cohorts contributed around 15.5% of the total size. Since the overlap between the two papers was limited, and in order not to discard valuable information, both papers were included in the meta-analysis and treated as if they were completely independent.

The main characteristics of included papers are summarized in Table 1. 

The papers were published between 1994 and 2020 and reported on studies conducted in Europe (n = 12), the USA (n = 7), Asia (n = 5), and Australia (n = 1). In terms of design, all were prospective cohort studies, except for Spencer et al. [25], Siezen et al. [31], and Tiemersma et al. [36], which were nested case–control studies, and Pietinen et al., whose study population consisted of subjects previously enrolled in a randomized controlled trial testing the effect of alpha-tocopherol and beta-carotene supplementation on lung cancer risk among male smokers [38]. The 25 studies included 2,228,377 individuals altogether, aged between 15 and 99 years at cohort inception, among which a total of 25,777 CRC cases were diagnosed during an average follow-up that varied between 4.8 and 14.9 across the studies. Seven and two studies included only women and men, respectively; the proportion of women ranged between 40.3% and 70.1% in the remaining sixteen papers. The breakdown of CRC cases into subsites was fully specified in seventeen studies; the studies by Gaard et al. and Bostick et al. included only colon cancer cases [39,40]. Information on food consumption was collected using food-frequency questionnaires in all studies except in Spencer et al. where 4-to-7-day diet diaries were used [25]. All included studies were of fair to very good quality, with the scores (assigned according to the NOS tool) ranging from the maximum allowed of 9 (for nine articles) to a minimum of 6 (for two articles) (Table S1).

The studies varied widely both in the way the exposure of interest was defined (e.g., (total) fish, fish and shellfish, (fish and) seafoods, etc.) and in the availability of risk estimates for specific subtypes of fish (e.g., fatty vs. lean, oily vs. non-oily, marine vs. fresh water, etc.) (Table S2). Dietary consumptions were reported by using as a unit of measure either the daily intake (mostly g/day) or the frequency of consumption (e.g., times or servings per week or month) (Table 1). 

**Figure 2:**
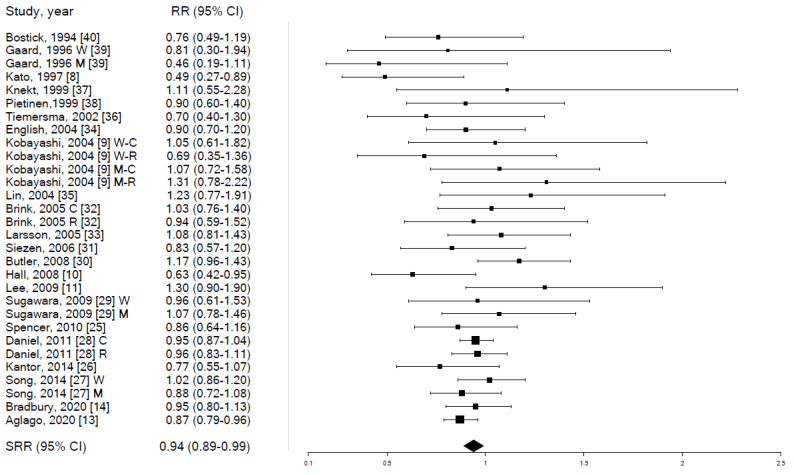
Forest plot for the association between fish consumption (highest vs. lowest category of consumption) and colorectal cancer risk. RR: relative risk. SRR: summary relative risk. CI: confidence intervals. W: RR among women. M: RR among men. C: RR for colon cancer. R: RR for rectal cancer.

In meta-analysis, individuals in the highest (vs. lowest) category of fish consumption had a mild yet statistically significant reduction in CRC risk (SRR 0.94, 95% CI 0.89–0.99, *p*-value 0.023), based on data from 22 independent studies, with negligible heterogeneity (I^2^ = 11.7%) and no evidence of publication bias (Egger’s test *p*-value = 0.955) (Table 2 and Table S3 and Figure 2). 

A comparable (in direction and strength) association emerged in stratified analyses (expect sex-stratified SRR for colon cancer risk, which was 1.02 among both women and men) but statistical significance was not achieved because of the lower number of available risk estimates (Table 2). In detail, the reduction in CRC risk for those in the highest category of fish consumption was slightly more evident among men (SRR 0.91) than women (SRR 0.95), while not differing by cancer site (SRR 0.94 for both colon and rectum) (Tables S4–S11) (Figures S1–S8). The association differed significantly (*p*-value 0.007) by country, as the SRR was 0.91 (95% CI 0.82–1.00) for studies conducted in North America, (n = 7), 0.90 (95% CI 0.84–0.97) for studies conducted in Europe (n = 10), and 1.12 (95% CI 0.98–1.27) in Asian studies (n = 4). 

In dose–response meta-analysis, a 50-g increment in the daily consumption of fish was associated with a statistically significant 4% reduction in CRC risk (SRR 0.96, 95% CI 0.92–0.99, *p*-value 0.021), based on data from seven independent studies, with no evidence of heterogeneity between studies (I^2^ = 0.0%) (Table 3). 

**Figure 3:**
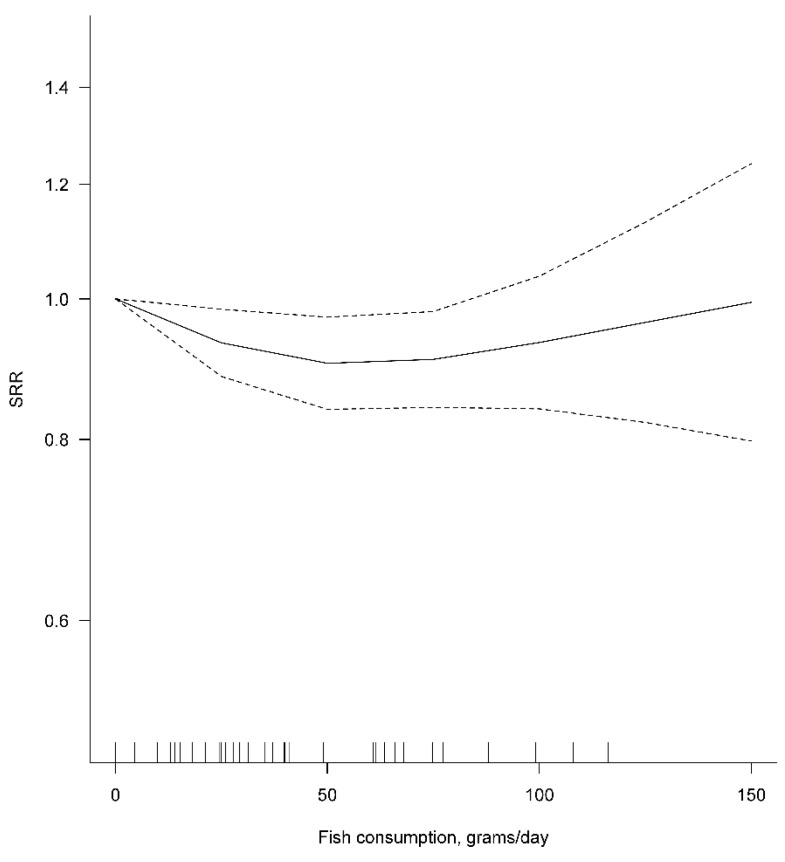
Non-linear dose–response meta-analysis for the association between fish consumption (g/day) and colorectal cancer risk. SRR: summary relative risk.

Results were similar in analyses stratified by sex or cancer site (Table 3). The results of the dose–response meta-analysis were heavily influenced by the single study of Aglago et al. [13], whose percentage study weight was 66.5% in the main analysis and ranged between 59.5% and 73.4% in stratified analysis. The *p*-value for deviation from linearity of the dose–response association was nearly significant (0.071) only for the main analysis. The visual inspection of the graph obtained by fitting a non-linear dose–response meta-analysis revealed that the decrease in CRC risk was observed only for the daily consumption of fish increasing up to 50 g/day and appeared to level off (albeit confidence intervals became increasingly wide because of limited data availability) above this value (Figure 3). 

### 3.2. Fish and Fish-Oil Intake and Colorectal Carcinogenesis in Animal Models 

The literature search resulted in 281 entries altogether: upon removing duplicates and checking inclusion criteria, a total of 25 articles were finally included in the review. The majority of preclinical experiments with rodents dealt with the effects of FO or pure ω-3 polyunsaturated fatty acids (PUFA) on colorectal carcinogenesis, while only a few studies were conducted by administering fish meat to the animals. 

In 1986, Reddy and Maruyama, using an experimental model in which colorectal carcinogenesis was chemically induced (with 1,2-dimethylhydrazine (DMH) or its metabolite azoxymethane (AOM)), first documented in rats that a diet containing FO as a source of fat (Menhaden oil) reduces colon tumorigenesis when compared with a diet containing the same amount of fat as corn oil (CO) which is rich in ω-6 PUFA [41] (Table 4 and Table S11). 

The same authors found that diets containing high levels of FO and low levels of CO were associated with fewer AOM-induced colon cancers compared with diets containing only CO [42]. Later investigations confirmed that Menhaden oil is effective in reducing both the initiation and post-initiation stages of carcinogenesis when compared to ω-6 PUFA-rich CO [44,45]. Studies were conducted in parallel in which pure ω-3 PUFA was administered instead of not-purified FO. Minoura et al. found that rats fed with eicosapentaenoic acid (EPA) had lower colon carcinogenesis and lower prostaglandin E2 (PGE2) levels than rats on a linoleic acid (LA) diet [43]. Takahashi et al. reported fewer DMH-induced microscopic preneoplastic lesions (aberrant crypt foci (ACF)) and protection against AOM-induced carcinogenesis among rats fed with pure docosahexaenoic acid (DHA) [46,47,48,49]. Subsequent studies confirmed the role of FO in preventing AOM-induced colon carcinogenesis through enhanced cell differentiation and apoptosis [50], particularly when compared to diets containing a mixture of lipids simulating the Western diet (lipids derived from saturated fats, peanut and corn oils) [51] and suggested that the effect could be mediated via reduced cyclooxygenase-2 (COX-2) activity and prostaglandin production. 

A beneficial effect of DHA was also observed in genetic models of intestinal carcinogenesis (Apc∆716 mice) [52] (Table 4 and Table S11). Likewise, an ω-3 PUFA ethyl ester-enriched fish oil concentrate was shown to oppose tumor growth in the small intestine (but not in the colon) in Apc-Min mice [53], and subsequent studies suggested that the protective effect could be mediated via antagonism with the production of arachidonic acid (a precursor of eicosanoids such as PGE2) [54]. More recently, highly purified EPA as free fatty acid (EPA-FFA) was observed to reduce COX-2 expression and cell proliferation and, eventually, prevent intestinal carcinogenesis in Apc-Min mice [55]. The potential of EPA-FFA in preventing colon carcinogenesis was later confirmed in a preclinical model mimicking CRC arising in the setting of inflammatory bowel disease (colitis-associated colorectal cancer (CAC)) [56].

Regarding colon cancer metastasis, Menhaden oil, in 1989, was first shown to be able to suppress the growth of a transplanted colon cancer cell line (CT-26 cells) and inhibit pulmonary colonization [59] (Table 5 and Table S13).

Similar results were seen in mice transplanted with a highly metastatic murine colon carcinoma cell line (Co 26 Lu) and fed with EPA and DHA [60], which were also effective in decreasing the number of lung metastasis via reduced metalloproteinase-2 and -9 activity [61]. Furthermore, EPA was also reported to inhibit the formation of liver metastatic foci of previously injected ACL-15 rat colon cancer cells by decreasing tumor cell proliferation and adhesion to the capillary bed [62]. Despite initial findings documenting adverse effects [63], ω-3 PUFA were reported to be able to prevent the development of colon cancer metastases in the liver of Wag-Rij rats transplanted with an established colon cancer cell line [64]. Subsequent experiments using EPA-FFA in the diet reported largely consistent results [65] and identified, among the possible mechanisms of action, a shift from PGE2 to PGE3 in tumor cells, as well as a reduced ERK signaling at the invasive edge of tumors. 

As already mentioned, only a few studies documented the effect of edible parts of fish on colon carcinogenesis (Table 4). The effect of salmon muscle was tested by Steppler et al. in A/J Min/+ mice, Apc mutated animals showing tumorigenesis not only in the small intestine but also in the colon [66]. Salmon-fed animals showed a slightly lower intestinal carcinogenesis when compared to a standard diet and to diets containing meat from terrestrial animals [57]. More recently, dietary supplementation with tuna muscle extract rich in selenoeine (an Se-protein with antioxidant activity present in the blood and muscle of tuna) was reported to decrease colon carcinogenesis in a colitis-associated model of CRC in mice [58]. 

## 4. Discussion

We conducted an up-to-date meta-analysis aiming to summarize the most recent available evidence about the association between fish consumption and CRC risk among humans. Twenty-five studies were included that encompassed a total of over 25,000 CRC cases arising from over 2.2 million individuals. The most important finding was the mild yet statistically significant reduction in CRC risk for individuals in the highest (vs. lowest) category of fish consumption, a finding that was made particularly trustworthy by the fair consistency of risk estimates across the studies and by the lack of evidence of publication bias. Analyses stratified by subsite showed that the effect was similar for colon and rectal cancer, the lower number of available studies for subsite-specific analyses being, therefore, the most likely explanation for the failure to achieve full statistical significance. Analyses stratified by sex also yielded comparable results to the main analysis in terms of both the direction and strength of the association, while geographical variability existed, whereby significant results emerged only in studies conducted in Europe and North America. The latter finding may partly be due to chance (only four of the included studies were conducted in Asia), but geographical diversity in terms of the genetic background and cooking methods of populations may also play a role; therefore, more research on the topic would be desirable to disentangle the importance of the different factors potentially at play. Finally, while based on only seven independent studies, the dose–response analysis showed a 4% decrease in CRC risk associated with a 50-g daily increase in fish consumption.

We then carried out a review of preclinical studies that reported on the link between the intake of fish and fish oil and colon carcinogenesis in animal models. While the diversity across the studies in terms of experimental methods advised against applying a formal meta-analytical approach to summarize the results, the reviewed studies were fairly consistent in suggesting a beneficial effect of fish- and fish oil-based diets against colorectal carcinogenesis and dissemination in rodents and identified several biological mechanisms potentially able to underlie this effect. 

The preventive effect of fish consumption on CRC risk observed in epidemiological studies can recognize several possible explanations. Partly, the association may be attributable to a replacement effect since those who eat more fish generally eat less red meat, whose causal link with colorectal carcinogenesis is well-known. Additionally, preferring fish instead of meat may be part of a generally healthier lifestyle encompassing other habits effective in preventing cancer [67,68]. However, prospective epidemiological studies are fairly consistent in detecting a reduction in CRC risk associated with fish consumption, which is, therefore, difficult to dismiss as mostly due to bias. In this regard, preclinical studies using animal models have been instrumental in helping to understanding the process of colorectal carcinogenesis and to give insights on how this may be affected by intrinsic and external factors such as diet, thus, providing support to the evidence stemming from epidemiological studies. Among animal models, both chemically-induced carcinogenesis and genetic models (the latter, mostly based on Apc mutations in mice and rats [69]) have been used to test the effect of dietary treatments, which are typically administered to the animals after the chemical induction of tumorigenesis or, in genetic models, starting from weaning. Depending on the duration of dietary treatment, either precursor lesions or straightforward tumors were used as a primary end-point of treatment efficacy. As stated before, the majority of animal experiments were carried out testing the effect of FO or its pure components (e.g., EPA and DHA) on colon carcinogenesis, while fish muscle consumption has not been thoroughly tested. This underlies the need to conduct studies in which animals are fed with fish, possibly using as a control group animals fed the same number of proteins and fat deriving from other animal muscles (i.e., beef or chicken) to resemble an actual human diet more closely. Notably, the majority of preclinical data documented a beneficial effect of FO and its pure compounds when compared with fats more common in the Western diet (like ω-6 or saturated fats), which emerged when focusing on CRC, its early phases (ACF or polyps), and even metastasis (although with some exceptions, e.g., Griffini et al.).

The reviewed studies highlighted some of the several biological mechanisms that may account for the protective effect of fish and FO against colorectal carcinogenesis. A detailed overview is beyond the scope of the present paper (interested readers may refer to existing reviews [5,6,70,71]), but some data deserve to be briefly described here. ω-3 PUFAs affect eicosanoids metabolism by inhibiting PGE_2_ production, EPA can function as a substrate for COXs to synthesize unique 3-series prostaglandin compounds (e.g., PGE_3_) less endowed with inflammatory action compared to 2-series prostaglandin compounds [72]. EPA and DHA produce lipid mediators endowed with pro-resolving, immunomodulatory, and anti-inflammatory properties (which may also explain their effect in preventing cardiovascular diseases) [6]. The molecular basis for the health benefits of ω-3 PUFA was also ascribed to the incorporation of these fatty acids into membrane phospholipids. Moreover, ω-3 PUFA do not enhance the luminal concentration of secondary bile acids (unlike saturated fats and ω-6 PUFA) and lower colon and liver activity of ornithine decarboxylase (ODC) and tyrosine-specific protein kinase (TPK), all of which are implicated in colon carcinogenesis [70]. Positive epigenetic effects have also been described together with interaction of ω-3 PUFA with nuclear receptors and transcription factors, thus, altering the proliferation, lipid metabolism, and apoptosis of cancer cells [5]. More recently, ω-3 PUFA was reported to be associated with higher intestinal microbial diversity, thus, improving host immune function and eventually halting the development of CRC [71,73].

ω-3 PUFA is also the nutrient contained in fish that was most extensively investigated in relation to CRC risk among humans, including in several prospective studies that mostly yielded results consistent with preclinical studies. In 2020, Kim et al. published a meta-analysis of twenty prospective studies which encompassed a total of 18,102 cases and over 1.3 million participants and found that ω-3 PUFA intake (from fish or FO supplements) was inversely associated with CRC risk [7]. In particular, linear increments by 0.1 g/day in the intake of EPA and DHA were associated with statistically significant 5% and 3% reductions, respectively, in CRC risk. Similar reviews for fish components other than ω-3 PUFA are warranted to reach a clearer understanding of the mechanisms underlying the observed protective effect of fish consumption against CRC risk. Like for every food, however, it is important to emphasize that the nutritional value of fish, and its association with human disease, is better evaluated on the basis of its consumption as a whole rather than as the effect of any single nutrient therein, both because the different nutrients may interact between one another in complex ways once they have entered the body, and because the food matrix affects how the food is digested and absorbed [3,74]. The finding of Kim et al.’s meta-analysis deserves attention, however, because it is consistent with data from animal models and confirms the role of ω-3 PUFA intake as an important (although not exclusive) mediator of the protective effect of fish consumption against CRC risk. 

The main strength of this paper lies in our having reviewed the available evidence from both preclinical and epidemiological studies with the aim of giving as broad an overview as possible on the role of fish in the prevention of CRC and on the possible mechanisms underlying this effect. The meta-analysis of prospective human studies takes advantage of a very large size and of a remarkable consistency of results across time and space: the studies were conducted in the USA, Europe, Australia, and Asia, and participants were followed up from as early as 1967 until as late as 2014. Results from highest-versus-lowest and dose–response analyses were consistent, heterogeneity among studies was low-to-negligible, and there was no evidence of publication bias, which further contributed to the reliability of our findings. Articles included in the review of preclinical studies were published over a period of more than three decades (1986–2018): in spite of the inevitable differences in study methods, the data were considerably consistent and provided valuable support to those originating from epidemiological studies, as well as many insights into underlying causal mechanisms. Our work also has some weaknesses that are important to acknowledge. A few papers may have been missed for having limited the search to only two databases (PUBMED/MEDLINE and EMBASE): however, the meticulous citation chaining of the papers included, the previously published reviews, the meta-analysis, and the lack of evidence for publication bias in meta-analysis suggest that the number of missed papers is unlikely to be high and their impact on the summary results minimal. Regarding the epidemiological studies, we merged results from studies that varied in terms of how the exposure of interest (“fish”) was defined (crustaceans and shellfish were included only in some studies, and a clear definition was sometimes missing) and measured (g/day or frequency of consumption). Moreover, the categories of fish consumption used to conduct the highest-versus-lowest analysis differed across studies, which is, however, usual in meta-analysis in the field of nutritional epidemiology. Furthermore, only a limited subset of studies (7 out of 25) contributed to the dose–response meta-analysis and summary results were heavily affected by a single study [13] which contributed around two-thirds of the statistical power of that analysis. Our meta-analysis does not suggest that any major disparity by sex exists in the association between fish consumption and CRC risk; however, only around half of the studies provided separate risk estimates for men and women, which may have curbed our ability to detect small differences in the strength of the association. With regard to studies on animal models, in addition to the aforementioned very large variability in study design, materials and methods, and to the use of fish meat (instead of fish oil) in only a very limited subset of studies, an important limitation is the fact that some of the potential biological mechanisms (e.g., modification of COX-2 activity, prostaglandins production, and others) were investigated in only a few studies; therefore, confirmation is needed to corroborate and extend current knowledge.

## 5. Conclusions

In conclusion, by jointly reviewing epidemiological studies among human populations and preclinical studies in animal models we found evidence that increasing fish consumption may effectively help inhibit colorectal carcinogenesis. CRC is a disease characterized by high incidence and mortality rates worldwide, so even a moderate reduction in its risk at the individual level (such as that achievable by increasing the consumption of fish, according to our findings) may lead to a major reduction in its disease burden at the population level. The consistent evidence from epidemiological and preclinical studies that fish consumption may be effective in preventing CRC should be taken into account when making dietary recommendations for cancer prevention. 

## Figures and Tables

**Table 1:** Main characteristics of articles included in the systematic review and meta-analysis on the association between fish consumption and colorectal cancer risk.

First Author, Year	Country	Study Name	Study Size ^(a)^	Men (%)	Age Range (at Cohort Inception)	Study Length	Follow-Up Time (Years)	Fish Consumption Assessment ^(b)^	No. Cases	Anatomic Site Distribution of Colorectal Cancers		
										Colon	Rectal	Ns or Other
Aglago, 2020 [13] ^(c)^	Europe	European Prospective Investigation into Cancer and Nutrition (EPIC)	476,160	29.9%	25–70	1992–ns	median 14.9	intake + frequency	6291	66.7%	33.3%	0.0%
Bradbury, 2020 [14]	UK	UK biobank study	475,581	46.0%	40–69	2006–2014	mean 5.7	intake + frequency	2609	66.8%	33.2%	0.0%
Kantor, 2014 [26]	USA	VITamins And Lifestyle cohort	68,109	49.0%	50–70	2001–2008	mean 6.7	frequency	488	63.7%	26.3%	10.0%
Song, 2014 [27]	USA	Nurses’ Health Study	76,386	0.0%	30–55	1976–2010	mean 14.8	intake	1469	76.9%	21.1%	2.0%
		Health Professionals Follow-up Study	47,143	100.0%	40–75	1986–2010			987	65.2%	21.8%	13.0%
Bamia, 2013 [22] ^(c)^	Europe	European Prospective Investigation into Cancer and Nutrition	480,308	30.0%	25–70	1992–2010	mean 11.6	intake	4355	63.2%	36.8%	0.0%
Daniel, 2011 [28]	USA	National Institutes of Health (NIH)-AARP Diet and Health Study	492,186	59.6%	50–71	1995–2006	mean 9.1	intake	7143	71.3%	26.4%	2.3%
Spencer, 2010 [25]	UK	UK Dietary Cohort Consortium	2575	45.9%	ns	1985–2006	ns	intake	579	ns	ns	100.0%
Murff, 2009 [24] ^(d)^	China	Shanghai Women’s Health Study	73,243	0.0%	40–70	1996–2007	ns	intake	396	ns	ns	100.0%
Lee, 2009 [11] ^(d)^	China	Shanghai Women’s Health Study	74,942	0.0%	45–65	1997–2005	mean 7.4	intake	394	59.9%	40.1%	0.0%
Sugawara, 2009 [29]	Japan	Ohsaki National Health Insurance Cohort Study	39,498	47.7%	40–79	1995–2003	mean 7.7	intake	566	59.5%	40.5%	0.0%
Butler, 2008 [30]	Singapore	Singapore Chinese Health Study	61,321	ns	45–74	1993–ns	mean 9.8	intake	961	61.5%	38.5%	0.0%
Hall, 2008 [10]	USA	Physicians’ Health Study	21,406	100.0%	ns	1982–2006	ns	frequency	500	77.6%	22.4%	0.0%
Engeset, 2007 [23] ^(c)^	Norway	The Norwegian Women and Cancer	64,168	0.0%	40–71	1991–2004	ns	intake	254	ns	ns	100.0%
Siezen, 2006 [31]	The Netherlands	Monitoring Project on Cardiovascular Disease Risk Factors + Diagnostisch Onderzoek Mammacarcinoom	160	ns	ns	1976–2003	ns	frequency	160	ns	ns	100.0%
Brink, 2005 [32]	The Netherlands	The Netherlands Cohort Study	2948	48.4%	55–69	1986–1993	mean 5.0	intake	608	73.7%	26.3%	0.0%
Larsson, 2005 [33]	Sweden	The Swedish Mammography Cohort	61,433	0.0%	40–75	1987–2003	mean 13.9	frequency	733	53.1%	31.4%	15.5%
English, 2004 [34]	Australia	The Melbourne Collaborative Cohort Study	37,112	39.5%	27–75	1990–2003	mean 9.0	frequency	451	62.5%	37.3%	0.2%
Kobayashi, 2004 [9]	Japan	The Japan Public Health Center-based prospective study	116,194	49.3%	40–69	1990–1999	ns	intake	705	ns	ns	100.0%
Lin, 2004 [35]	USA	Women’s Health Study	37,547	0.0%	45–ns	1993–2003	mean 8.7	frequency	202	80.2%	19.8%	0.0%
Tiemersma, 2002 [36]	The Netherlands	Monitoring Project on Cardiovascular Disease Risk Factors	102	54.9%	20–59	1987–1998	mean 8.5	frequency	102	61.8%	ns	ns
Knekt, 1999 [37]	Finland	Cohort assembled within the Mobile Health Clinic of the Social Insurance Institution	9985	52.8%	15–99	1967–1990	ns	intake	189	38.6%	ns	61.4%
Pietinen, 1999 [38]	Finland	The Alpha-Tocopherol, Beta-Carotene Cancer Prevention Study	27,111	100.0%	50–69	1985–1995	mean 8.0	intake	185	ns	ns	ns
Kato, 1997 [8]	USA	New York University Women’s Health Study	14,727	0.0%	34–65	1985–1994	mean 7.1	frequency	100	84.0%	16.0%	0.0%
Gaard, 1996 [39]	Norway	Cohort assembled within the Norwegian National Health Screening Service	50,535	50.1%	20–54	1977–ns	mean 11.4	frequency	143	100.0%	0.0%	0.0%
Bostick, 1994 [40]	USA	Iowa Women’s Health Study	35,216	0.0%	55–69	1986–1990	mean 4.8	frequency	212	100.0%	0.0%	0.0%

^(a)^ Cohort size or (for nested case–control studies) number of CRC cases. ^(b)^ Food intake was ex-pressed in g/day except in Daniel et al. 2001 (g/1000 kcal/day). Frequency was expressed as times, servings, or meals per week or month depending on the study. ^(c)^ Bamia et al. [22] was based on a shorter follow-up of the EPIC study than in Aglago et al. [13]. Engeset et al. [23] was based on one of the country-specific EPIC cohorts. From the studies by Bamia et al. and Engeset et al. we con-sidered only the results not available in Aglago et al. ^(d)^ The results from Murff et al. [24] were used only when they were not available in Lee et al. [11], which was based on the same cohort.

**Table 2:** Meta-analysis of studies for the association between fish consumption (highest vs. lowest category of consumption) and colorectal cancer risk, overall and stratified by tumor site and sex.

	N Studies	SRR	Lower 95% CI	Upper 95% CI	*p*-Value	I^2^
Colorectal cancer	22	0.94	0.89	0.99	0.023	11.7%
women	13	0.95	0.87	1.05	0.310	13.9%
men	10	0.91	0.82	1.01	0.088	27.2%
Colon cancer	15	0.94	0.88	1.01	0.089	12.9%
women	7	1.02	0.88	1.19	0.763	12.5%
men	4	1.02	0.85	1.22	0.866	0.0%
Rectal cancer	13	0.94	0.87	1.03	0.173	0.0%
women	5	0.99	0.79	1.26	0.966	0.0%
men	3	0.90	0.57	1.43	0.663	63.2%

SRR: summary relative risk. CI: confidence intervals.

**Table 3:** Dose–response meta-analysis of studies for the association between fish consumption (linear increment by 50 g/day) and colorectal cancer risk, overall and stratified by tumor site and sex.

	N Studies	SRR (for an Increase by 50 g/day)	Lower 95% CI	Upper 95% CI	*p*-Value	I^2^	*p*-Value for Non-Linearity
Colorectal cancer	7	0.96	0.92	0.99	0.021	0.0%	0.071
women	5	0.95	0.90	1.01	0.078	0.0%	0.880
men	6	0.97	0.92	1.02	0.188	0.0%	0.280
Colon cancer	6	0.96	0.92	1.01	0.140	0.0%	0.991
Rectal cancer	6	0.95	0.89	1.02	0.174	0.0%	0.248

SRR: summary relative risk. CI: confidence intervals.

**Table 4:** Effects of fish or its components on different animal models of colorectal cancer.

Experimental Model	Dietary Intervention and Duration of Treatment	Effects on Carcinogenesis	Reference
AOM-induced rats	Diets containing Menhaden or Corn oils	Reduction in CRC in rats fed Menhaden Oil.	Reddy, 1986 [41]
-+AOM-induced rats	Different levels of FO or CO in the diet.	Reduction in CRC in rats fed high FO.	Reddy, 1988 [42]
AOM-induced rats	Diets containing EPA or LA	Reduction in colon carcinogenesis in the EPA group.	Minoura, 1988 [43]
AOM-induced mice	Commercial preparations of FO or CO.	Reduction in dysplastic areas and carcinogenesis in the FO group.	Deschner, 1990 [44]
AOM-induced rats	Diets containing FO and/or CO.	Reduction in colon carcinogenesis in rats fed high FO.	Reddy, 1991 [45]
DMH-induced rats	DHA (0.7 mL by gavage) 5 times a wk.	Reduction in ACF.	Takahashi, 1993 [46]
AOM-induced rats	DHA (0.7 mL of by gavage) twice a wk.	Slight non-significant reduction of CRC.	Takahashi, 1994 [47]
		Significant reduction in ACF.	
AOM-induced rats	DHA (1 mL by gavage) 5 times a wk.	Significant reduction in ACF and in CRC.	Takahashi, 1997a [48]
2-amino-1-methyl-6-phenylimidazo [4,5-b]pyridine (PhIP)-induced rats	DHA (1 mL by gavage) 5 times a wk.	Reduction in ACF.	Takahashi, 1997b [49]
AOM-induced rats	Diet supplemented with FO or CO; cellulose or pectin also tested.	FO reduced CRC.	Chang, 1998 [50]
AOM-induced rats	FO vs. HFML.	Reduction in carcinogenesis and ACF in rats fed FO.	Rao, 2001 [51]
Apc^∆716^ mice of both sexes	Diet with 3% DHA for 7 wks.	Reduction in SI polyps, only in female mice fed DHA.	Oshima, 1995 [52]
Min mice mutated in Apc	Diet containing a commercial FO preparation.	Reduction in SI tumors in treated mice.	Paulsen, 1997 [53]
Min mice mutated in Apc	Diets containing EPA (1.5%) or different PUFA for 8 wks.	Reduction in SI tumors in the EPA group.	Petrik, 2000 [54]
Min mice mutated in Apc	EPA-FFA fed for 12 wks.	Reduction in polyp number in SI and colon.	Fini, 2011 [55]
Mice treated with AOM/DSS to induce CAC	EPA-FFA in the diet tested in the initiation and post-initiation phases.	Reduction in tumorigenesis	Piazzi, 2014 [56]
A/J Min/+ mice of both sexes	Salmon compared with beef or chicken muscles fed from weaning for 10 wks.	Salmon muscle decreased tumor load and size in the SI.	Steppler, 2017 [57]
Mice treated with AOM-DSS to induce CAC	Tuna muscle extract rich in Selenoeine fed for 14 wks.	Reduction in carcinogenesis.	Masuda, 2018 [58]

**Table 5:** Effects of fish or its components on different animal models of colorectal cancer metastasis.

Experimental Model	Dietary Intervention and Duration of Treatment	Effects on Colon Cancer and Metastasis	Reference
Mice inoculated with CT-26 tumor cells	Diets containing fish or safflower oils fed for 30 days before and after CT-26 transplantation.	Fish oil (FO) reduced tumor growth and pulmonary colonization.	Cannizzo, 1989 [59]
Mice s.c. implanted with Co 26 Lu tumor cells	Diets containing EPA and DHA during and after cancer cell transplantation.	Inhibition of tumor growth and decrease in lung metastatic nodules.	Iigo, 1997 [60]
Mice s.c. implanted with Co 26 Lu tumor cells	Diets containing EPA, DHA, LA, or oleic acid (OA) from day 5 for a total of 3 wks after cell implantation.	EPA, DHA, and OA reduced metastasis. Tumor cells treated with DHA showed a very low potential for lung colony formation when injected i.v.	Suzuki, 1997 [61]
Rats inoculated with ACL-15 tumor cells	Diets containing EPA, LA, or PA. ACL-15 tumor cells inoculated at 6 wks and rats sacrificed at 9 wks.	EPA reduced metastatic foci in liver. EPA and PA diet groups had smaller liver metastatic foci.	Iwamoto,1998 [62]
Rats injected (via portal vein) with CC531 tumor cells	Diets containing FO or safflower oil for 3 wks before CC531 inoculation until sacrifice after 1 or 3 wks.	FO increased metastasis (number and size) at 1 wk after implantation; both FO and the safflower oil diets increased metastasis 3 wks after implantation.	Griffini, 1998 [63]
Rats injected (into the spleen) with CC531 tumor cells	Diets containing PUFA or coconut oil in the diet 3 days before and 28 days after CC 531 injection.	Reduction in tumor growth in the PUFA group.	Gutt, 2007 [64]
Mice injected (into the spleen) with MC-26 tumor cells	Diet containing EPA-FFA. Feeding 2 wks before and after cell injection.	EPA-FFA administration caused a reduced MC-26 liver tumor burden.	Hawcroft, 2012 [65]

## Supplementary Materials

The following supporting information can be downloaded at: https://www.mdpi.com/article/10.3390/cancers14030640/s1, Figure S1: Forest plot for the association between fish consumption (highest vs. lowest category of consumption) and colorectal cancer risk among women; Figure S2: Forest plot for the association between fish consumption (highest vs. lowest category of consumption) and colorectal cancer risk among men; Figure S3: Forest plot for the association between fish consumption (highest vs. lowest category of consumption) and colon cancer risk; Figure S4: Forest plot for the association between fish consumption (highest vs. lowest category of consumption) and colon cancer risk among women; Figure S5: Forest plot for the association between fish consumption (highest vs. lowest category of consumption) and colon cancer risk among men; Figure S6: Forest plot for the association between fish consumption (highest vs. lowest category of consumption) and rectal cancer risk; Figure S7: Forest plot for the association between fish consumption (highest vs. lowest category of consumption) and rectal cancer risk among women; Figure S8: Forest plot for the association between fish consumption (highest vs. lowest category of consumption) and rectal cancer risk among men; Table S1: Quality assessment (using the Newcastle–Ottawa Scale tool) of the articles included in the meta-analysis; Table S2: Exact definition (as reported in the paper text) of the exposures that were studied in relation to colorectal, colon, or rectal cancer risk in each included study. Risk estimates entered in meta-analysis models were those marked with the asterisk (*); Table S3: Studies and corresponding relative risk estimates included in the meta-analysis of the association between fish consumption (highest vs. lowest category of consumption) and colorectal cancer risk; Table S4: Studies and corresponding relative risk estimates included in the meta-analysis of the association between fish consumption (highest vs. lowest category of consumption) and colon cancer risk; Table S5: Studies and corresponding relative risk estimates included in the meta-analysis of the association between fish consumption (highest vs. lowest category of consumption) and rectal cancer risk; Table S6: Studies and corresponding relative risk estimates included in the meta-analysis of the association between fish consumption (highest vs. lowest category of consumption) and colorectal cancer risk among women; Table S7: Studies and corresponding relative risk estimates included in the meta-analysis of the association between fish consumption (highest vs. lowest category of consumption) and colon cancer risk among women; Table S8: Studies and corresponding relative risk estimates included in the meta-analysis of the association between fish consumption (highest vs. lowest category of consumption) and rectal cancer risk among women; Table S9: Studies and corresponding relative risk estimates included in the meta-analysis of the association between fish consumption (highest vs. lowest category of consumption) and colorectal cancer risk among men; Table S10: Studies and corresponding relative risk estimates included in the meta-analysis of the association between fish consumption (highest vs. lowest category of consumption) and colon cancer risk among men; Table S11: Studies and corresponding relative risk estimates included in the meta-analysis of the association between fish consumption (highest vs. lowest category of consumption) and rectal cancer risk among men; Table S12: Experiments with fish and its components on animal models of colorectal cancer; Table S13: Experiments with fish or its components on different animal models of colorectal cancer metastasis.

## Abbreviations

ACF: Aberrant Crypt Foci. AOM: Azoxymethane. AOM/DSS: Azoxymethane/Dextran Sodium Sulfate. APC: Adenomatous Polyposis Coli. CAC: Colitis-Associated Colorectal cancer. CI: Confidence Intervals. CO: Corn Oil. COX-2: Cyclossigenase-2. CRC: Colorectal Cancer. DHA: Docosahexaenoic Acid. DMH: 1,2-Dimethylhydrazine. EPA: Eicosapentaenoic acid. EPA-FFA: Eicosapentaenoic Acid as Free Fatty Acid. EPIC: European Prospective Investigation into Cancer and Nutrition. ERK: Extracellular Signal-Regulated Kinase. FFA: Free Fatty Acid. FO: Fish Oil. GCO: Global Cancer Observatory. HFML: High-Fat diet containing Mixed Lipids. LA: Linoleic Acid. LogRR: Log Relative Risk. MOOSE: Guidelines for Meta-Analyses and Systematic Reviews of Observational Studies. NCC: Nested Case–Control studies. NOS: Newcastle–Ottawa Scale. OA: Oleic Acid. ODC: Ornithine Decarboxylase. PA: Palmitic Acid. PGE_2_: Prostaglandin E_2._ PGE_3_: Prostaglandin E_3._ PhIP: 2-amino-1-methyl-6-phenylimidazo [4,5-b]pyridine. PUFA (ω-3): Long chain ω-3 Polyunsturated Fatty Acids. RCT: Randomized Clinical Trials. RR: Relative Risk. SI: Small Intestine. SRR: Summary Risk Ratio. TPK: Tyrosine-specific Protein Kinase. WCRF: World Cancer Research Fund.
