# Supplementary material for: Fish Consumption and Colorectal Cancer Risk: Meta-Analysis of Prospective Epidemiological Studies and Review of Evidence from Animal Studies
Source: Cancers (Basel). 2022 Jan 27;14(3):640. doi: 10.3390/cancers14030640 (PMC8833371; doi:10.3390/cancers14030640)
Supplement: Supplementary file 1 [file cancers-14-00640-s001.zip › cancers-1537155-supplementary.pdf]

Figure S1. Forest plot for the association between fish consumption (highest vs. lowest category of consumption) and colorectal cancer risk among women.

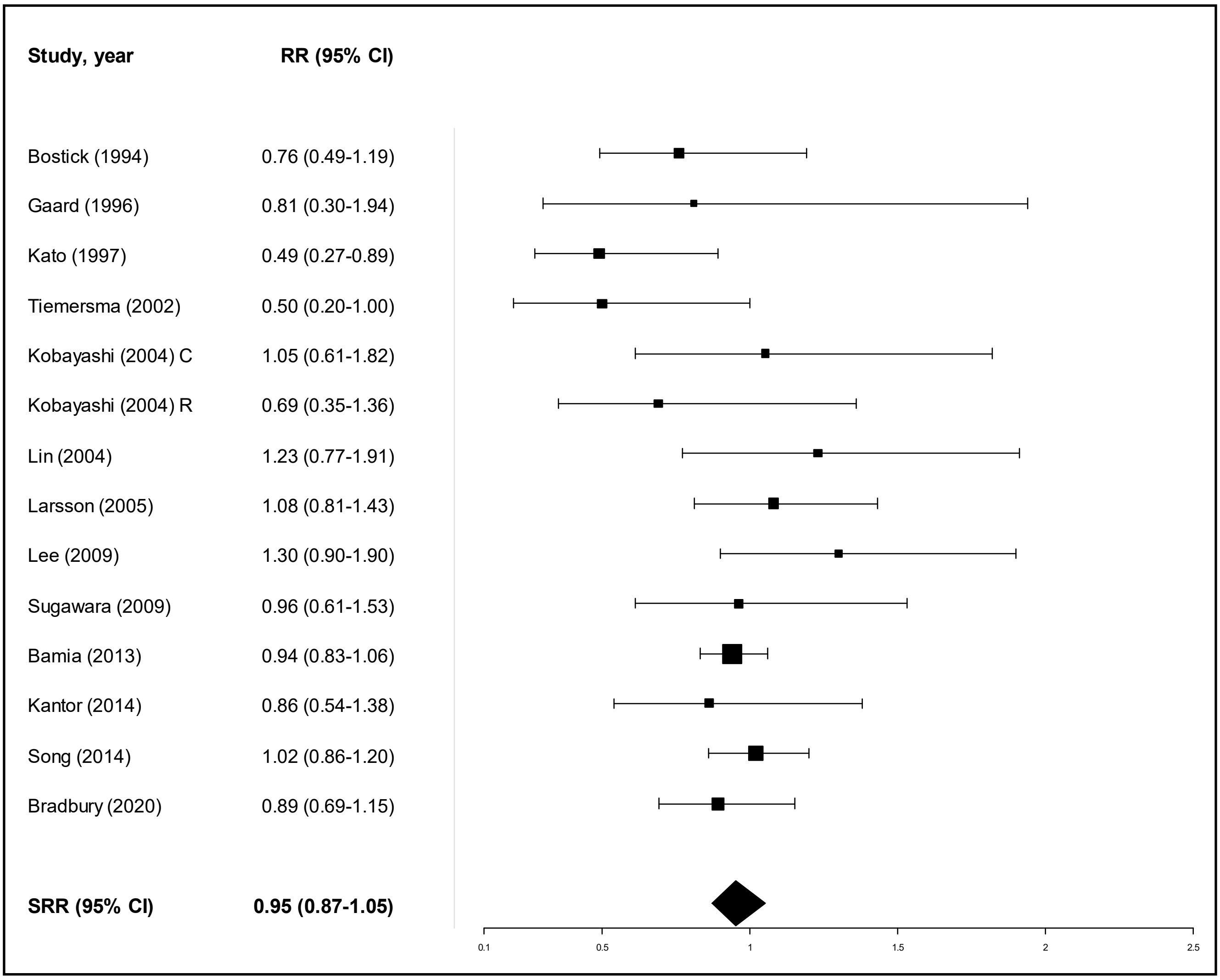

RR: relative risk. SRR: summary relative risk. CI: confidence intervals.

C: RR for colon cancer. R: RR for rectal cancer.

Figure S2. Forest plot for the association between fish consumption (highest vs. lowest category of consumption) and colorectal cancer risk among men.

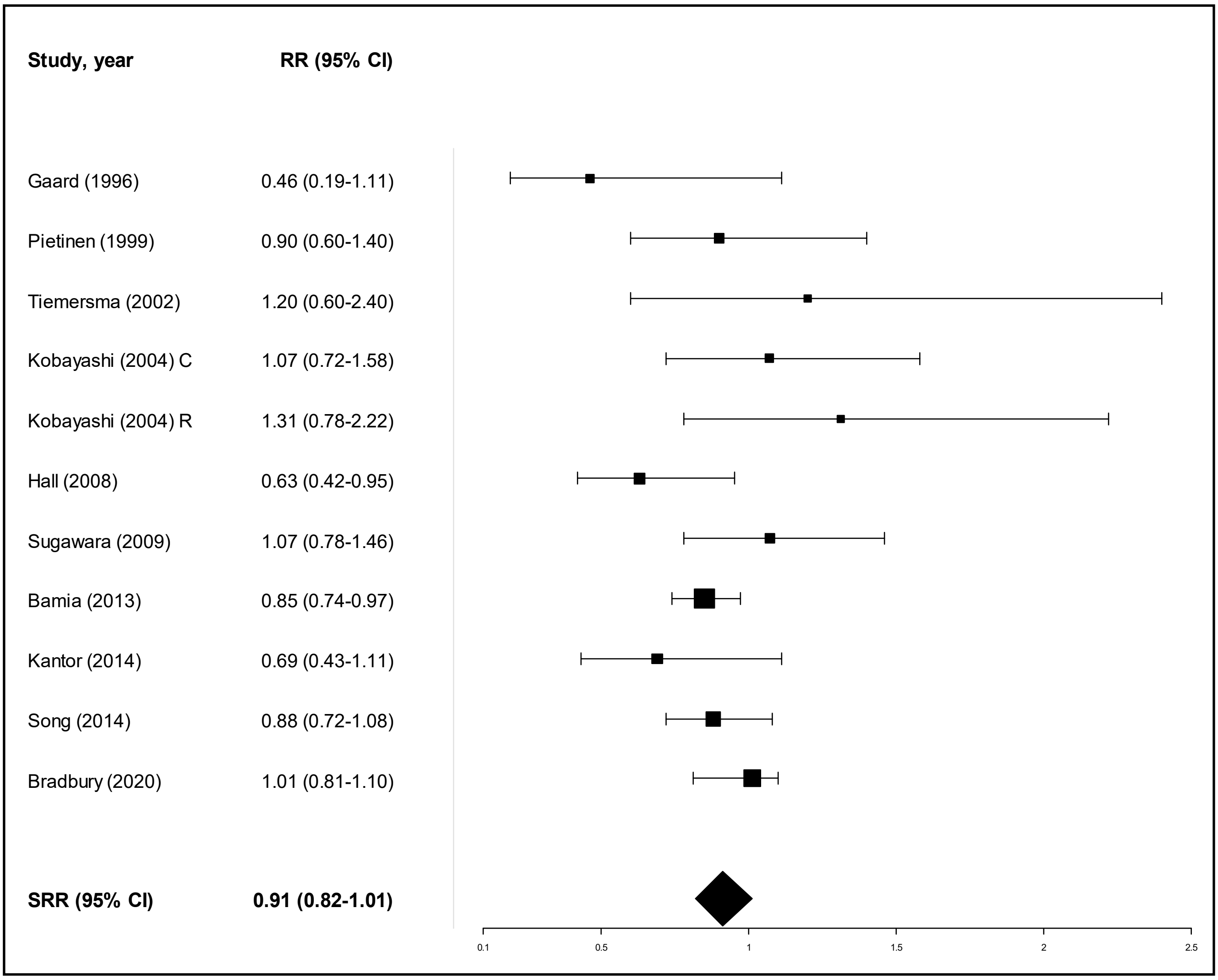

RR: relative risk. SRR: summary relative risk. CI: confidence intervals.

C: RR for colon cancer. R: RR for rectal cancer.

Figure S3. Forest plot for the association between fish consumption (highest vs. lowest category of consumption) and colon cancer risk.

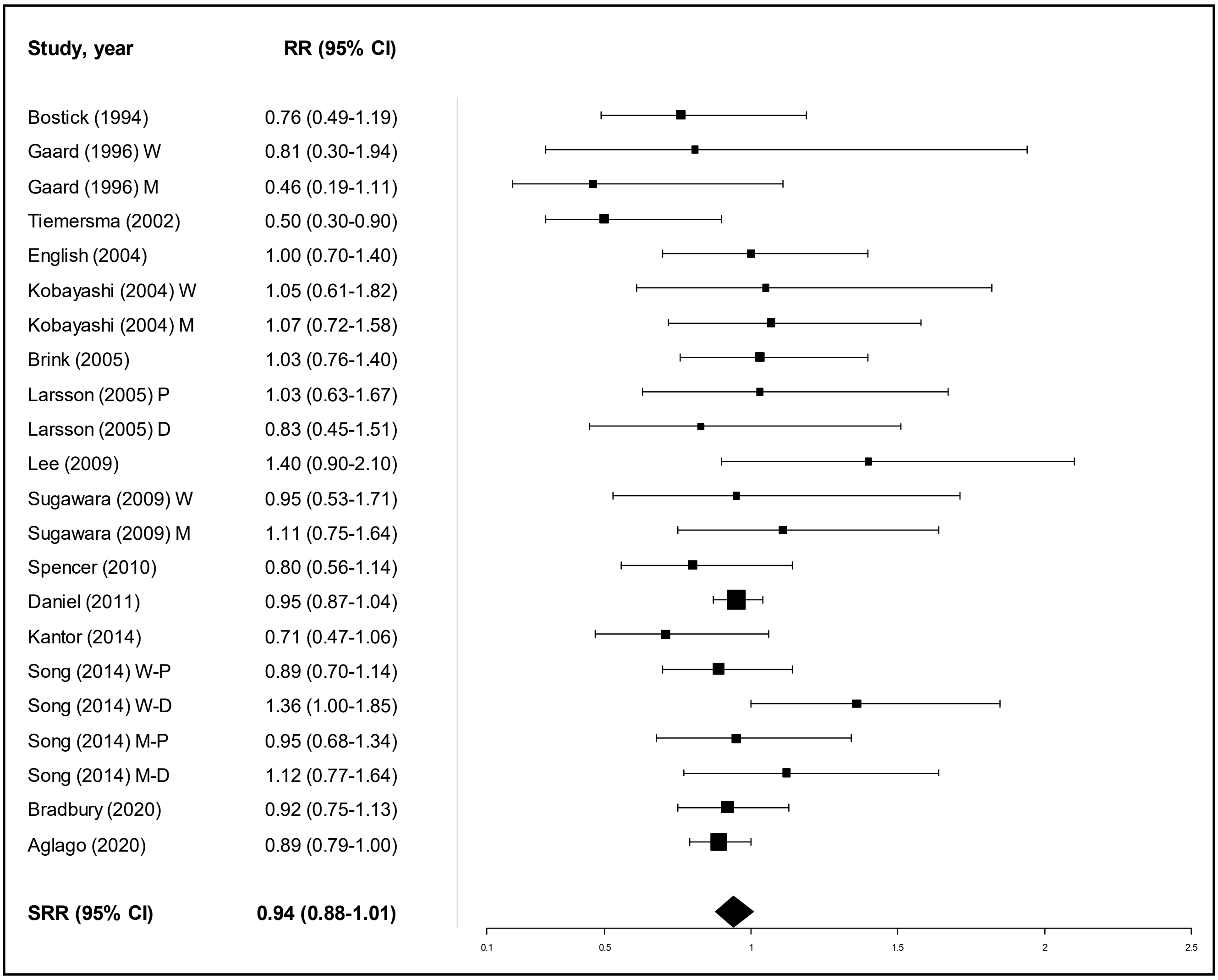

RR: relative risk. SRR: summary relative risk. CI: confidence intervals.

W: RR among women. M: RR among men.

P: RR for proximal colon cancer. D: RR for distal colon cancer.

Figure S4. Forest plot for the association between fish consumption (highest vs. lowest category of consumption) and colon cancer risk among women.

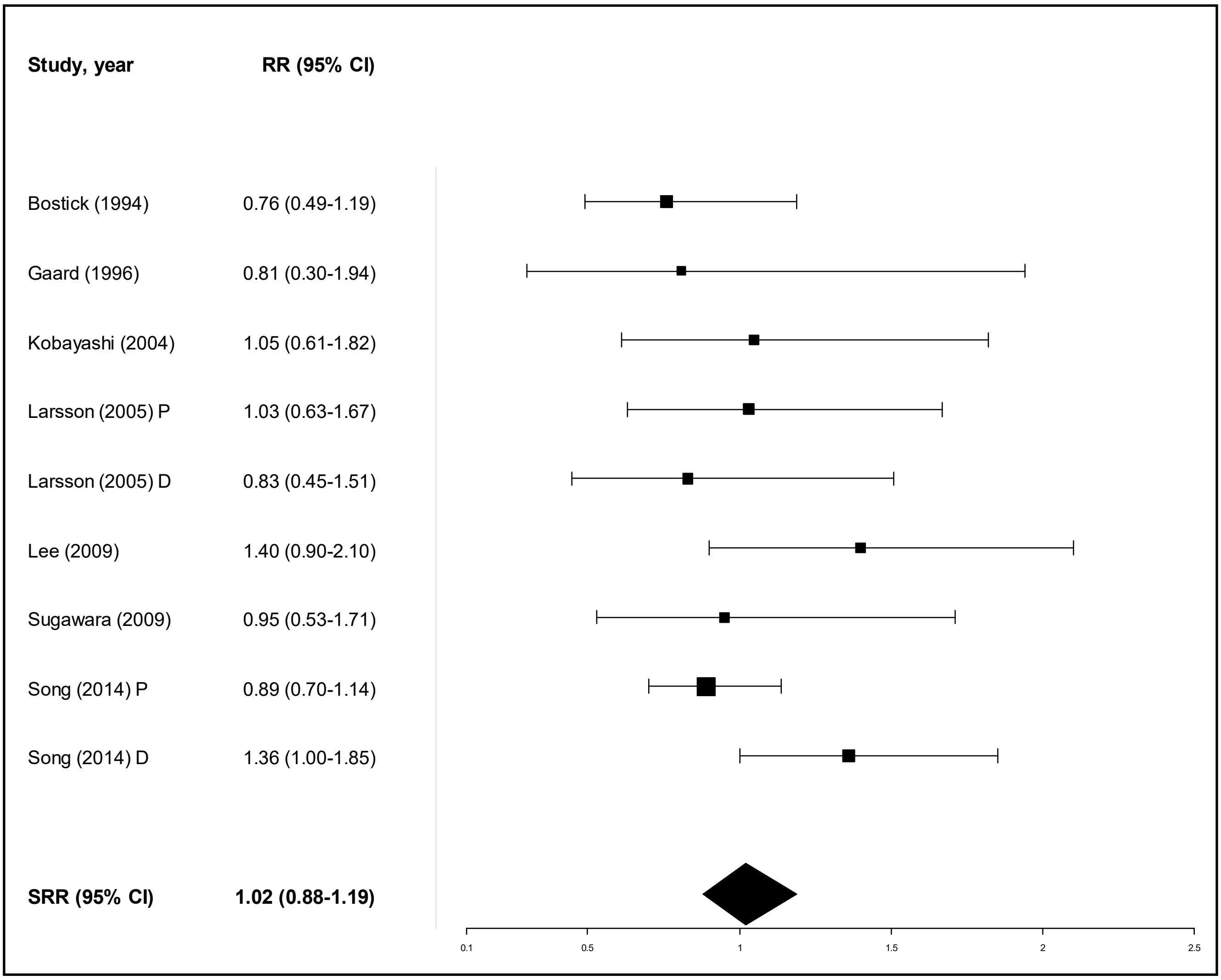

RR: relative risk. SRR: summary relative risk. CI: confidence intervals.

P: RR for proximal colon cancer. D: RR for distal colon cancer.

Figure S5. Forest plot for the association between fish consumption (highest vs. lowest category of consumption) and colon cancer risk among men.

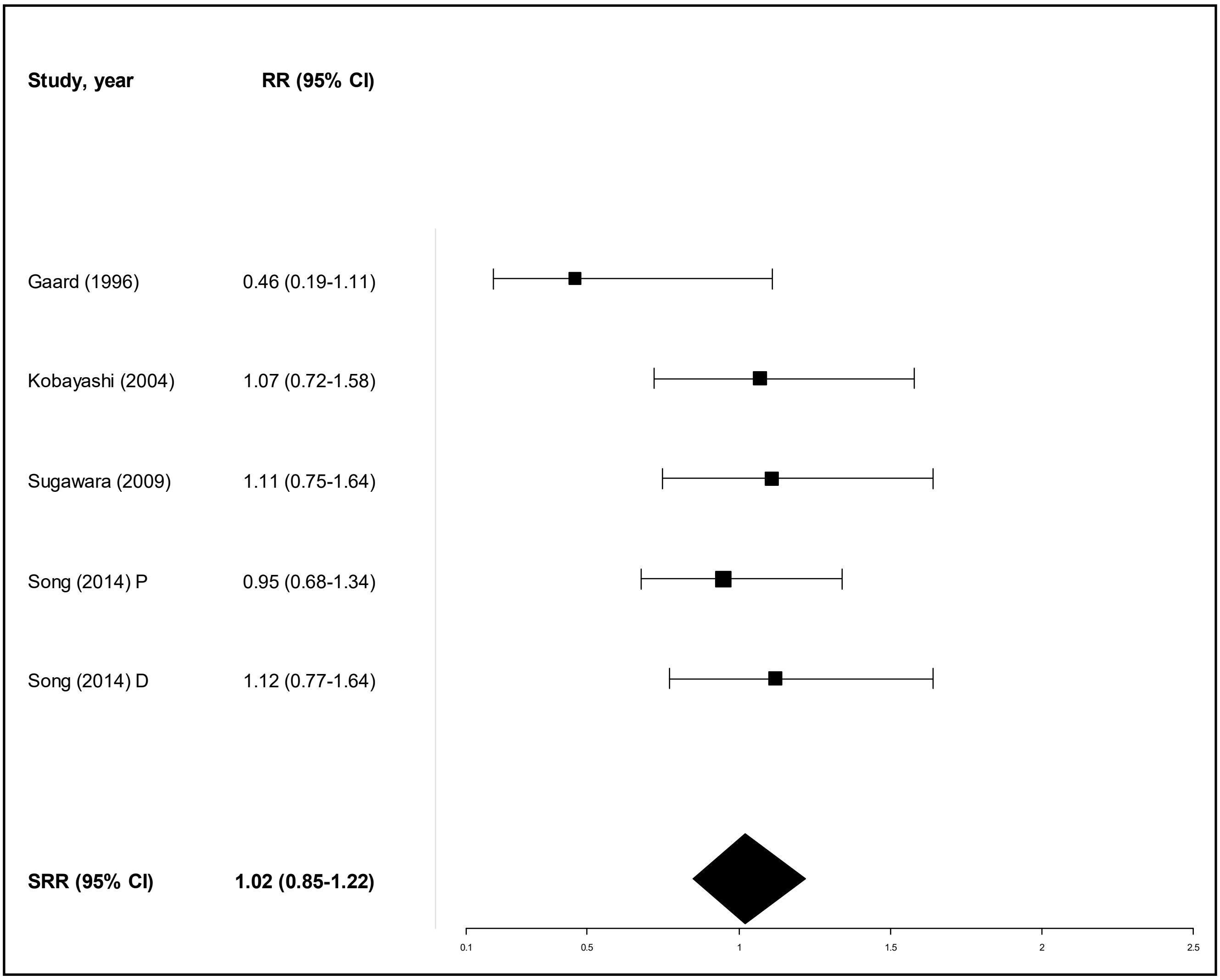

RR: relative risk. SRR: summary relative risk. CI: confidence intervals.

P: RR for proximal colon cancer. D: RR for distal colon cancer.

Figure S6. Forest plot for the association between fish consumption (highest vs. lowest category of consumption) and rectal cancer risk.

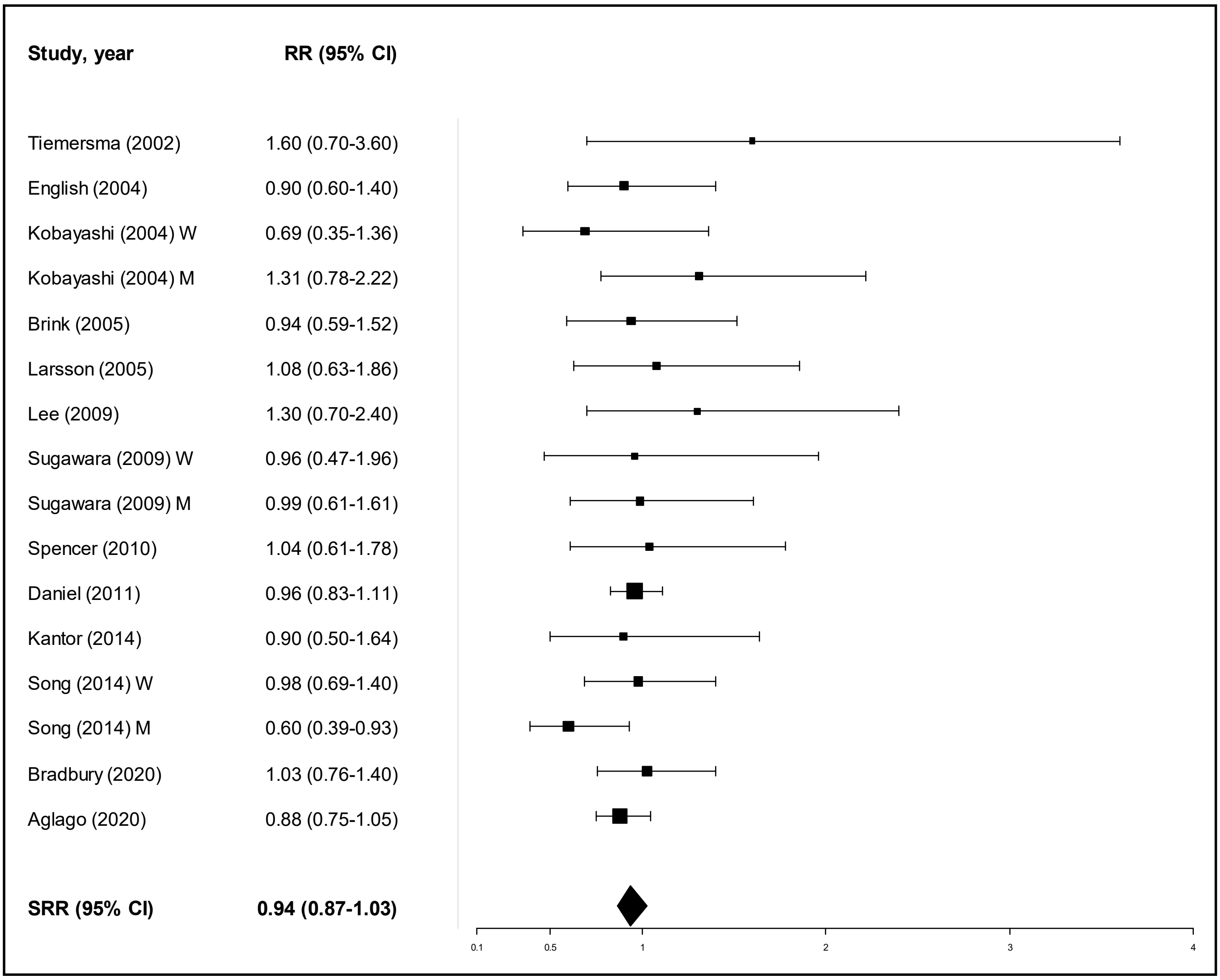

RR: relative risk. SRR: summary relative risk. CI: confidence intervals.

W: RR among women. M: RR among men.

Figure S7. Forest plot for the association between fish consumption (highest vs. lowest category of consumption) and rectal cancer risk among women.

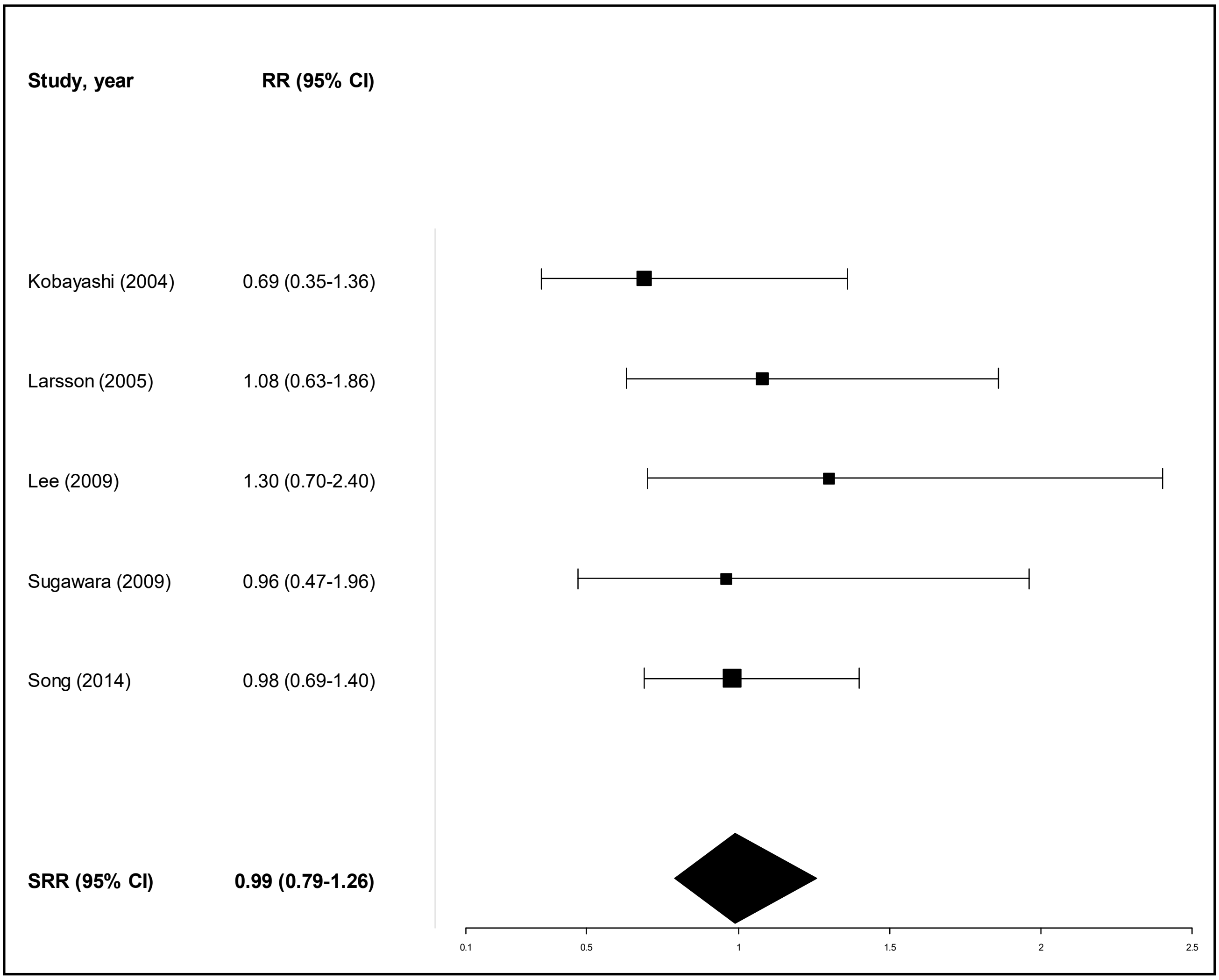

RR: relative risk. SRR: summary relative risk. CI: confidence intervals.

Figure S8. Forest plot for the association between fish consumption (highest vs. lowest category of consumption) and rectal cancer risk among men.

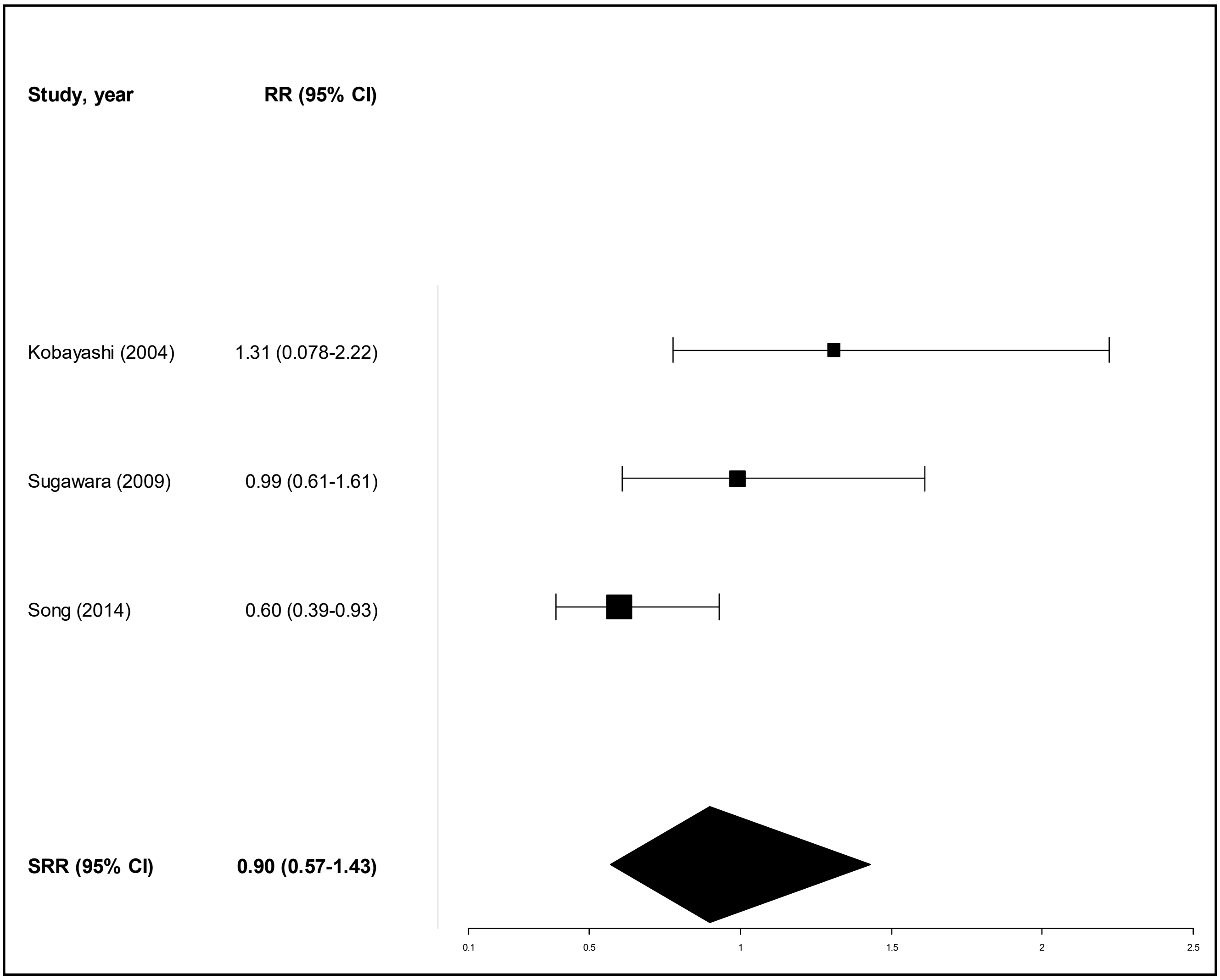

RR: relative risk. SRR: summary relative risk. CI: confidence intervals.

Table S1. Quality assessment (using the Newcastle–Ottawa Scale tool) of the articles included in the meta-analysis;.

**Newcastle-Ottawa quality assessment scale**	**Aglago, 2020**	**Bradbury, 2019**	**Seizen, 2006**	**Kantor, 2014**	**Song, 2014**	**Bamia, 2013**	**Daniel, 2011**	**Spencer, 2010**	**Lee, 2009**	**Murff, 2009**	**Sugawara, 2009**	**Butler, 2008**	**Hall, 2008**	**Engeset, 2007**	**Brink, 2005**	**Larsson, 2005**	**English, 2004**	**Kobayashi, 2004**	**Lin, 2004**	**Tiemersma, 2004**	**Knekt, 1999**	**Pietinen, 1999**	**Kato, 1997**	**Gaard, 1996**	**Bostick, 1994**
**Selection**																									
1) Representativeness of the exposed cohort	1	1	1	1	1	1	1	1	1	0	1	1	0	1	1	0	1	1	0	1	1	0	0	1	0
2) Selection of the non-exposed cohort	1	1	1	0	1	1	1	1	1	0	1	1	1	1	1	1	1	1	1	1	1	1	1	1	1
3) Ascertainment of exposure	1	1	1	1	1	1	1	1	1	1	1	1	1	1	1	1	1	1	1	1	1	1	1	1	1
4) Demonstration that outcome of interest was not present at start of study	1	1	0	1	1	1	1	1	1	0	0	1	1	1	0	1	1	0	1	1	0	1	1	1	1
**Comparability**																									

1) Comparability of cohorts on the basis of the design or analysis	2	2	2	2	2	2	2	2	2	2	2	2	2	2	2	2	2	2	2	2	2	2	2	2	2
**Outcome**																									
1) Assessment of outcome	1	1	1	1	1	1	1	1	1	1	1	1	1	1	1	1	1	1	1	1	1	1	1	1	1
2) Was follow-up long enough for outcomes to occur	1	0	1	0	1	1	1	1	1	1	1	1	1	1	1	1	1	1	1	1	1	1	1	1	0
3) Adequacy of follow-up of cohorts	1	1	1	1	0	1	1	1	1	1	1	1	1	1	0	1	1	1	1	1	0	1	1	1	0
	**9**	**8**	**8**	**7**	**8**	**9**	**9**	**9**	**9**	**6**	**8**	**9**	**8**	**9**	**7**	**8**	**9**	**8**	**8**	**9**	**7**	**8**	**8**	**9**	**6**

Table S2. Exact definition (as reported in the paper text) of the exposures that were studied in relation to colorectal, colon, or rectal cancer risk in each included study. Risk estimates entered in meta-analysis models were those marked with the asterisk (*).

First author, year	Exposure	Definition (from paper text)
**Aglago, 2020**	total fish	fish + fish products (including fatty and lean fish but excluding fish oil supplements)
	fatty fish	fat > 4%/weight (e.g. salmon)
	lean fish	fat ≤ 4%/weight (e.g. cod)
	shellfish	e.g. prawn
	total fish + shellfish *	as above
**Bradbury, 2020**	total fish *	oily + non oily fish
	oily fish	ns
	non-oily fish	ns
**Kantor, 2014**	dark fish *	i.e. fresh tuna + salmon
**Song, 2014**	fish *	canned tuna + dark meat fish + other fish + shrimp + lobster + scallops + fish cakes + fish sticks
**Bamia, 2013**	fish *	fish + seafood
**Daniel, 2011**	fish *	all types of finfish + shellfish (breaded/deep fried fish or stick fish, other fish/seafood not fried) + canned tuna
**Spencer, 2010**	white fish *	all non-fatty fish, not including shellfish
	fatty fish	salmon, herring, fresh tuna + other oily fish
**Murff, 2009**	total fish *	ns
**Butler, 2008**	fish *	ns
**Lee, 2009**	total fish *	marine + fresh water fish
	marine fish	ns
	fresh water fish	ns
	eel	cholesterol-rich fish
	shrimp	cholesterol-rich fish
	shellfish	cholesterol-rich fish
	salted fish	cooking method
**Sugawara, 2009**	total fish *	fresh fish + fish products (fish paste made from boiled white fish meat as kamaboko or chikuwa) + dried or salted fish
**Hall, 2008**	fish *	fish or shellfish: dark fish (mackerel, salmon, sardines, bluefish, swordfish) + canned tuna fish + shrimp + lobster + scallops + other fish
**Engeset, 2007**	total fish *	whole fish, fish products, crustaceans, roe and fish liver
	lean fish	fish with less than 4% fat (e.g. cod, haddock, saithe)
	fatty fish	fish with 4% fat and above (e.g. salmon, trout, herring, mackerel)

	fish products	fish fingers (coated in bread crumbs), preserved fish (e.g. canned mackerel, smoked salmon, canned tuna), fish casseroles and minced fish products (e.g. fish balls, fish cakes)
	poached lean fish	ns
	fried lean fish	ns
**Siezen, 2006**	fish *	ns
**Brink, 2005**	fish + shellfish *	ns
**Larsson, 2005**	fish *	lean fish + fatty fish (salmon, mackerel, sardines and herring)
**English, 2004**	fish *	steamed/grilled/baked/fried/smoked fish + canned fish (tuna + salmon + sardines)
**Kobayashi, 2004**	fish (cohort 1) *	fresh fish + dried fish + salted fish + fish roe + fermented fish products
	fish (cohort 2) *	fresh fish + dried fish + salted fish + fermented fish products + small fry + fish paste + canned fish
**Lin, 2004**	fish + other seafood *	fish + canned tuna + shrimp
**Tiemersma, 2002**	fish *	ns
**Knekt, 1999**	smoked and salted fish	ns
	fish (other) *	ns
**Pietinen, 1999**	fish *	ns
**Kato, 1997**	fish + shellfish *	ns
**Gaard, 1996**	fish meals *	poached, fried or processed fish
**Bostick, 1994**	seafood *	ns

ns: not specified

Table S3. Studies and corresponding relative risk estimates included in the meta-analysis of the association between fish consumption (highest vs. lowest category of consumption) and colorectal cancer risk.

Author, year	Exposure	Unit of measure	Highest vs. lowest (ref) category	RR	95% CI
Bostick, 1994 [(a)]	seafood	servings/week	> 2.5 vs. < 1	0.76	0.49-1.19
Gaard, 1996 [(a)] [(b)]	total fish	times/week	≥ 5 vs. ≤ 2	0.81	0.30-1.94
	total fish	times/week	≥ 5 vs. ≤ 3	0.46	0.19-1.11
Kato, 1997 [(c)]	fish + shellfish	ns	4th vs. 1st quartile	0.49	0.27-0.89
Knekt, 1999	fish (other)	intake	4th vs. 1st quartile	1.11	0.55-2.28
Pietinen, 1999 [(d)]	total fish	intake (g/day)	4th vs. 1st quartile	0.90	0.60-1.40
Tiemersma, 2002	fish	times/month	> 4 vs. 0-1	0.70	0.40-1.30
English, 2004	total fish	times/week	≥ 2.4 vs. < 1.4	0.90	0.70-1.40
Kobayashi, 2004 [(e)]	total fish	intake (g/day)	4th vs. 1st quartile	1.05	0.61-1.82
				1.07	0.72-1.58
				0.69	0.35-1.36
				1.31	0.78-2.22
Lin, 2004 [(c)]	fish + seafood	servings/day	5th vs. 1st quintile	1.23	0.77-1.91
Brink, 2005 [(e)]	total fish	intake (g/day)	4th vs. 1st quartile	1.03	0.76-1.40
				0.94	0.59-1.52
Larsson, 2005 [(c)]	fish	servings/week	≥ 2.0 vs. < 0.5	1.08	0.81-1.43
Siezen 2006	total fish	frequency	high vs. low fish consumption	0.83	0.57-1.20
Butler, 2008	seafood	servings/week	> 2.5 vs. < 1.0	1.17	0.96-1.43
Hall, 2008 [(d)]	fish	frequency	≥ 5 vs. <1.0	0.63	0.96-1.43
Lee, 2009 [(c)]	total fish	intake (g/day)	≥ 74 vs. < 20	1.30	0.90-1.90
Sugawara, 2009 [(b)]	total fish	intake (g/day)	≥ 81.4 vs. < 26.6	0.96	0.61-1.53
			≥ 96.4 vs. < 26.2	1.07	0.78-1.46
Spencer, 2010	white fish	intake (g/day)	≥ 30 vs. < 1	0.86	0.64-1.16
Daniel, 2011 [(f)]	total fish	g/1000 kcal	5th vs. 1st quintile	0.95	0.87-1.04
				0.96	0.83-1.11
Kantor, 2014	dark fish (salmon + tuna)	servings/week	≥ 0.80 vs. none	0.77	0.55-1.07
Song, 2014 [(b)]	total fish	intake (g/day)	≥ 40 vs. < 15	1.02	0.86-1.20
			≥ 46 vs. < 16	0.88	0.72-1.08
Bradbury, 2020	total fish	times/week	≥ 3 vs. < 1	0.95	0.80-1.13
Aglago, 2020	total fish + shellfish	intake (g/day)	> 56.1 vs. < 10.4	0.87	0.79-0.96
**Summary RR**				**0.94**	**0.89-0.99**

[(a)] This study only included colon cancer cases as study outcome.

[(b)] This study reported separate RR estimates for women (upper row) and men (lower row).

[(c)] This study included only women.

[(d)] This study included only men.

[e] This study reported separate RR estimates for (from up to bottom row): colon cancer among women, colon cancer among men, rectal cancer among women, and rectal cancer among men.

[f] This study reported separate RR estimates for colon cancer (upper row) and rectal cancer (lower row).

RR: relative risk. CI: confidence intervals. ns: not specified.

Table S4. Studies and corresponding relative risk estimates included in the meta-analysis of the association between fish consumption (highest vs. lowest category of consumption) and colon cancer risk.

Author, year	Exposure	Unit of measure	Highest vs. lowest (ref) category	RR	95% CI
Bostick, 1994 [(a)]	seafood	servings/week	> 2.5 vs. < 1	0.76	0.49-1.19
Gaard, 1996 [(b)]	total fish	times/week	≥ 5 vs. ≤ 2	0.81	0.30-1.94
			≥ 5 vs. ≤ 3	0.46	0.19-1.11
Tiemersma, 2002	fish	times/month	> 4 vs. 0-1	0.50	0.30-0.90
English, 2004	total fish	times/week	≥ 2.4 vs. < 1.4	1.00	0.70-1.40
Kobayashi, 2004 [(b)]	total fish	intake (g/day)	4th vs. 1st quartile	1.05	0.61-1.82
				1.07	0.72-1.58
Brink, 2005	total fish	intake (g/day)	4th vs. 1st quartile	1.03	0.76-1.40
Larsson, 2005 [(a) (c)]	fish	servings/week	≥ 2.0 vs. < 0.5	0.83	0.45-1.51
				1.03	0.63-1.67
Lee, 2009 [(a)]	total fish	intake (g/day)	≥ 74 vs. < 20	1.40	0.90-2.10
Sugawara, 2009 [(b)]	total fish	intake (g/day)	≥ 81.4 vs. < 26.6	0.95	0.53-1.71
			≥ 96.4 vs. < 26.2	1.11	0.75-1.64
Spencer, 2010	white fish	intake (g/day)	≥ 30 vs. < 1	0.80	0.56-1.14
Daniel, 2011	total fish	g/1000 kcal	5th vs. 1st quintile	0.95	0.87-1.04
Kantor, 2014	dark fish (salmon + tuna)	servings/week	≥ 0.80 vs. none	0.71	0.47-1.06
Song, 2014 [(d)]	total fish	intake (g/day)	≥ 40 vs. < 15	1.36	1.00-1.85
				0.89	0.70-1.14
			≥ 46 vs. < 16	1.12	0.77-1.64
				0.95	0.68-1.34
Bradbury, 2020	total fish	times/week	≥ 3 vs. < 1	0.92	0.75-1.13
Aglago, 2020	total fish + shellfish	intake (g/day)	> 56.1 vs. < 10.4	0.89	0.79-1.00
**Summary RR**				**0.95**	**0.89-1.01**

[(a)] This study included only women.

[(b)] This study reported separate RR estimates for women (upper row) and men (lower row).

[(c)] This study reported separate RR estimates for distal colon (upper row) and proximal colon (lower row).

[(d)] This study reported separate RR estimates for (from up to bottom row) distal colon among women, proximal colon among women, distal colon among men, and proximal colon among men.

RR: relative risk. CI: confidence intervals.

Table S5. Studies and corresponding relative risk estimates included in the meta-analysis of the association between fish consumption (highest vs. lowest category of consumption) and rectal cancer risk.

Author, year	Exposure	Unit of measure	Highest vs. lowest (ref) category	RR	95% CI
Tiemersma, 2002	fish	times/month	> 4 vs. 0-1	1.60	0.70-3.60
English, 2004	total fish	times/week	≥ 2.4 vs. < 1.4	0.90	0.60-1.40
Kobayashi, 2004 [(a)]	total fish	g/day	4th vs. 1st quartile	0.69	0.35-1.36
				1.31	0.78-2.22
Brink, 2005	total fish	g/day	4th vs. 1st quartile	0.94	0.59-1.52
Larsson, 2005 [(b)]	fish	servings/week	≥ 2.0 vs. < 0.5	1.08	0.63-1.86
Lee, 2009 [(b)]	total fish	g/day	≥ 74 vs. < 20	1.30	0.70-2.40
Sugawara, 2009 [(a)]	total fish	g/day	≥ 81.4 vs. < 26.6	0.96	0.47-1.96
			≥ 96.4 vs. < 26.2	0.99	0.61-1.61
Spencer, 2010	white fish	g/day	≥ 30 vs. < 1	1.04	0.61-1.78
Daniel, 2011	total fish	g/1000 kcal	5th vs. 1st quintile	0.96	0.83-1.11
Kantor, 2014	dark fish (salmon and tuna)	servings/week	≥ 0.8 vs. 0.0	0.90	0.50-1.64
Song, 2014 [(a)]	total fish	g/day	≥ 40 vs. < 15	0.98	0.69-1.40
			≥ 46 vs. < 16	0.60	0.39-0.93
Bradbury, 2020	total fish	times/week	≥ 3 vs. < 1	1.03	0.76-1.40
Aglago, 2020	total fish and shellfish	g/day	> 56.1 vs. < 10.4	0.88	0.75-1.05
**Summary RR**				**0.94**	**0.87-1.02**

[(a)] This study reported separate RR estimates for women (upper row) and men (lower row).

[(b)] This study included only women.

RR: relative risk. CI: confidence intervals.

Table S6. Studies and corresponding relative risk estimates included in the meta-analysis of the association between fish consumption (highest vs. lowest category of consumption) and colorectal cancer risk among women.

Author, year	Exposure	Unit of measure	Highest vs. lowest (ref) category	RR	95% CI
Bostick, 1994 [(a)]	seafood	servings/week	>2.5 vs. <1	0.76	0.49-1.19
Gaard, 1996 [(a)]	fish	meals/week	≥5 vs. ≤2	0.81	0.30-1.94
Kato, 1997	fish and shellfish	intake (NS)	4th vs. 1st quartile	0.49	0.27-0.89
Tiemersma, 2002	fish	times/month	>4 vs. ≤1	0.50	0.20-1.00
Kobayashi, 2004 [(b)]	fish	g/day	4th vs. 1st quartile	1.05	0.61-1.82
				0.69	0.35-1.36
Lin, 2004	fish and other seafood	servings/day	5th vs. 1st quintile	1.23	0.77-1.91
Larsson, 2005	fish	servings/week	≥2 vs. <0.5	1.08	0.81-1.43
Lee, 2009	fish	g/day	≥74 vs. <20	1.30	0.90-1.90
Sugawara, 2009	fish	g/day	≥81.4 vs. ≤26.6	0.96	0.61-1.53
Bamia 2013	fish	g/day	3rd vs. 1st tertile	0.94	0.83-1.06
Kantor, 2014	dark fish (salmon and tuna)	servings/week	≥0.8 vs. 0.0	0.86	0.54-1.38
Song, 2014	fish	g/day	≥40 vs. <15	1.02	0.86-1.20
Bradbury, 2020	fish	times/week	≥3 vs. <1	0.89	0.69-1.15
**Summary RR**				**0.95**	**0.87-1.05**

[(a)] This study only included colon cancer cases as study outcome.

[(b)] This study reported separate RR estimates for colon cancer (upper row) and rectal cancer (lower row).

RR: relative risk. CI: confidence intervals.

Table S7. Studies and corresponding relative risk estimates included in the meta-analysis of the association between fish consumption (highest vs. lowest category of consumption) and colon cancer risk among women.

Author, year	Exposure	Unit of measure	Highest vs. lowest (ref) category	RR	95% CI
Bostick, 1994	seafood	servings/week	> 2.5 vs. < 1	0.76	0.49-1.19
Gaard, 1996	total fish	times/week	≥ 5 vs. ≤ 2	0.81	0.30-1.94
Kobayashi, 2004	total fish	intake (g/day)	4th vs. 1st quartile	1.05	0.61-1.82
Brink, 2005	total fish	intake (g/day)	4th vs. 1st quartile	1.03	0.76-1.40
Larsson, 2005 [(a)]	fish	servings/week	≥ 2.0 vs. < 0.5	0.83	0.45-1.51
				1.03	0.63-1.67
Lee, 2009	total fish	intake (g/day)	≥ 74 vs. < 20	1.40	0.90-2.10
Sugawara, 2009	total fish	intake (g/day)	≥ 81.4 vs. < 26.6	0.95	0.53-1.71
Song, 2014 [(a)]	total fish	intake (g/day)	≥ 40 vs. < 15	1.36	1.00-1.85
				0.89	0.70-1.14
**Summary RR**				**1.02**	**0.88-1.19**

[(a)] This study reported separate RR estimates for distal colon (upper row) and proximal colon (lower row).

RR: relative risk. CI: confidence intervals.

Table S8. Studies and corresponding relative risk estimates included in the meta-analysis of the association between fish consumption (highest vs. lowest category of consumption) and rectal cancer risk among women.

Author, year	Exposure	Unit of measure	Highest vs. lowest (ref) category	RR	95% CI
Kobayashi, 2004	total fish	g/day	4th vs. 1st quartile	0.69	0.35-1.36
Larsson, 2005	fish	servings/week	≥ 2.0 vs. < 0.5	1.08	0.63-1.86
Lee, 2009	total fish	g/day	≥ 74 vs. < 20	1.30	0.70-2.40
Sugawara, 2009	total fish	g/day	≥ 81.4 vs. < 26.6	0.96	0.47-1.96
Song, 2014	total fish	g/day	≥ 40 vs. < 15	0.98	0.69-1.40
**Summary RR**				**0.99**	**0.79-1.26**

RR: relative risk. CI: confidence intervals.

Table S9. Studies and corresponding relative risk estimates included in the meta-analysis of the association between fish consumption (highest vs. lowest category of consumption) and colorectal cancer risk among men.

Author, year	Exposure	Unit of measure	Highest vs. lowest (ref) category	RR	95% CI
Gaard, 1996 [(a)]	total fish	meals/week	≥ 5 vs. ≤ 2	0.46	0.19-1.11
Pietinen, 1999	total fish	g/day	4th vs. 1st quartile	0.90	0.60-1.40
Tiemersma, 2002	fish	times/month	>4 vs. 0-1	1.20	0.60-2.40
Kobayashi, 2004 [(b)]	total fish	g/day	4th vs. 1st quartile	1.07	0.72-1.58
				1.31	0.78-2.22
Hall, 2008	fish	frequency	≥ 5 vs. < 1	0.63	0.42-1.95
Sugawara, 2009	total fish	g/day	≥ 96.4 vs. < 26.2	1.07	0.78-1.46
Bamia 2013	fish	g/day	3rd vs. 1st tertile	0.85	0.74-0.97
Kantor, 2014	dark fish (salmon and tuna)	servings/week	≥ 0.8 vs. 0.0	0.69	0.43-1.11
Song, 2014	total fish	g/day	≥ 46 vs. < 16	0.88	0.72-1.08
Bradbury, 2020	total fish	times/week	≥ 3 vs. < 1	1.01	0.81-1.10
**Summary RR**				**0.91**	**0.82-1.01**

[(a)] This study only included colon cancer cases as study outcome.

[(b)] This study reported separate RR estimates for colon cancer (upper row) and rectal cancer (lower row).

RR: relative risk. CI: confidence intervals.

Table S10. Studies and corresponding relative risk estimates included in the meta-analysis of the association between fish consumption (highest vs. lowest category of consumption) and colon cancer risk among men.

Author, year	Exposure	Unit of measure	Highest vs. lowest (ref) category	RR	95% CI
Gaard, 1996	total fish	times/week	≥ 5 vs. ≤ 3	0.46	0.19-1.11
Kobayashi, 2004	total fish	intake (g/day)	4th vs. 1st quartile	1.07	0.72-1.58
Sugawara, 2009	total fish	intake (g/day)	≥ 96.4 vs. < 26.2	1.11	0.75-1.64
Song, 2014 [(a)]	total fish	intake (g/day)	≥ 46 vs. < 16	1.12	0.77-1.64
				0.95	0.68-1.34
**Summary RR**				**1.02**	**0.85-1.22**

[(a)] This study reported separate RR estimates for distal colon (upper row) and proximal colon (lower row).

RR: relative risk. CI: confidence intervals.

Table S11. Studies and corresponding relative risk estimates included in the meta-analysis of the association between fish consumption (highest vs. lowest category of consumption) and rectal cancer risk among men.

Author, year	Exposure	Unit of measure	Highest vs. lowest (ref) category	RR	95% CI
Kobayashi, 2004	total fish	g/day	4th vs. 1st quartile	1.31	0.78-2.22
Sugawara, 2009	total fish	g/day	≥ 96.4 vs. < 26.2	0.99	0.61-1.61
Song, 2014	total fish	g/day	≥ 46 vs. < 16	0.60	0.39-0.93
**Summary RR**				**0.90**	**0.57-1.43**

RR: relative risk. CI: confidence intervals.

Table S12. Experiments with fish and its components on animal models of colorectal cancer.

Experimental model	Dietary intervention and duration of treatment	Effects on carcinogenesis	Additional parameters measured	Reference
AOM-induced male F344 rats	Diets containing Menhaden Oil [22.5% (w/w) + CO 1% (w/w)] or CO [23.5% (w/w)]. Rats sacrificed 34 wks after the last AOM treatment.	Reduction in CRC in rats fed high content of FO, although this group also showed a significantly lower body weight than CO fed rats.	Food consumption in all dietary groups (during 12 and 22 wks).<br>Colon endoscopy in 4 animals/group at 20 and 30 wks after the last AOM injection.	Reddy, 1986
AOM-induced male F344 rats	Diets containing different levels of FO or CO.<br>Rats sacrificed 38 wks after the last AOM injection.	Reduction in CRC in rats fed high FO diets compared to those fed high CO.	Dose related inhibition of colonic mucosa ODC activity in the animals fed FO. Significant incorporation of ω-3 PUFA in the colonic and tumour microsomal fraction.	Reddy, 1988
AOM-induced male Donryu rats	4.7% (w/w) EPA in the diet + 0.3% (w/w) LA *vs* 5% (w/w) LA.<br>Rats sacrificed 15 wks after the last AOM injection.	Reduction in tumour incidence and multiplicity in the colon.<br>Rats fed EPA diet didn't show any tumours in the SI.	Lower levels of PGE_2 in tumours from EPA rats.	Minoura, 1988
AOM-induced female CF1 mice	Commercial preparation of FO vs CO administered in a sub-chronic (8 wks) and chronic experiments (50 wks). Mice sacrificed 48 wks after the first AOM injection.	Reduction in dysplastic areas (sub-chronic experiment) and in carcinogenesis (chronic experiments) compared with CO.	Amelioration of epithelial cell proliferation parameters in mice fed FO.	Deschner, 1990
AOM-induced male F344 rats	Menhaden oil and CO as sources of ω-3 PUFA and ω-6 PUFA, respectively. Rats sacrificed 42 wks after AOM treatment.	Significant colon carcinogenesis reduction in rats fed high Menhaden oil at both the initiation and post initiation phases of CRC.	Positive effects observed at both initiation and /or post-initiation phases of CRC.	Reddy, 1991
DMH-induced male F344 rats	DHA administered by gavage (0.7 mL of DHA vs water), 5 times a wk for 4, 8 and 12 wks. Rats sacrificed 4, 8, 12 wks after the first DMH injection.	Reduction of ACF (number and multiplicity) at all-time points sampled.	DHA treatment increased plasma levels of DHA and EPA and decreased arachidonic acid (AA) plasma level to about a one-half that in the control group.	Takahashi, 1993
AOM-induced male F344 rats	DHA administered by gavage (0.7 mL of DHA *vs* water), twice a wk. Rats sacrificed 4, 8 and 12 wks (ACF determination) or 36 wks (tumour determination) after the first AOM injection.	Slight, non-significant reduction of CRC by DHA.<br>Significant reduction of ACF in DHA-treated group.	DHA treatment increased the plasma levels of DHA and EPA and decreased those of AA, triglyceride and total cholesterol.	Takahashi, 1994
AOM-induced male F344 rats	DHA administered by gavage (1 mL of DHA *vs* water) 5 times a wk. Rats	Reduction of AOM-induced ACF and in the number of	DHA treatment significantly increased the DHA and EPA plasma levels and decreased AA level. The PGE_2	Takahashi, 1997a

	sacrificed 4, 12, 36 wks after the first AOM injection.	tumours in the colon and rectum by DHA treatment.	level at wk 36 was decreased by DHA treatment.	
2-amino-1-methyl-6-phenylimidazo [4,5-b]pyridine (PhIP)-induced male F344 rats	DHA or water (1 mL) administered by gavage 5 times a wk 4 h prior to PhIP treatment from day 1 for 2 wks and then further treated until wk 4 or 12. Four wks after the first treatment with PhIP, animals were sacrificed.	Reduction in ACF by DHA.	At 4 wks the PhIP-DNA adduct levels in the colon of rats from the PhIP/DHA group were approximately two thirds of the PhIP group value.	Takahashi, 1997b
AOM-induced male Sprague-Dawley rats	FO *vs* CO. The effect of dietary fiber (cellulose or pectin) was also tested. Rats sacrificed at 18 and 36 wks after the last AOM injection.	Fish oil caused a lower tumor incidence than CO.	No effect of FO on proliferative activity, but FO increased the degree of differentiation and apoptosis compared with CO.	Chang, 1998
AOM-induced male F344 rats	FO *vs* HFML, a mix of lipids, mimicking Western diets. Rats sacrificed 8, 23, and 38 wks after the last AOM injection.	Lower carcinogenesis and ACF in rats fed fish oil *vs* HFML.	Higher apoptosis in the tumours from rats on fish oil when compared to those on HFML.	Rao, 2001
Apc$^{\Delta 716}$ mouse of both sexes	AIN7-76A purified diet containing 3% DHA for 7 wks. Mice sacrificed at 10 wks of age.	Reduction in the SI polyps, but only in female. No effect in male mice.		Oshima, 1995
Min mice mutated in *Apc* of both sexes	ω-3 PUFA ethyl ester enriched FO concentrate (K85), up to 2.5% (w/w) in the diet. From wk 1 until wk 18 after birth.	Reduction in SI tumours.	No effect on colon tumours or in ACF.	Paulsen, 1997
Min male mice mutated in *Apc*	AIN-93G diet supplemented with 1,5 % (w/w) of EPA or different PUFA. Feeding of the diets for 8 wks. Sacrifice at 100 days of age.	Reduction in tumorigenesis in the SI (no effect in the colon).	Reduction in intestinal AA content and the levels of PGE in the colon of mice EPA group.	Petrik, 2000
Min male mice mutated in *Apc*	Purified EPA as FFA (EPA-FFA) fed for 12 wks.	Reduction in polyp number through the intestine (SI and colon).	Reduction of lipid peroxidation; EPA replaces AA in the membranes; reduction of proliferative activity and increase in apoptosis.	Fini, 2010

C57BL/6J male mice treated with AOM/DSS to induce colitis and cancer	1% (w/w) EPA-FFA in the diet and tested in the initiation and post-initiation phases. Mice sacrificed 11 wks after AOM injection.	Reduction in polyp incidence, multiplicity tumor size.	Increase in apoptosis, decrease in proliferation activity. EPA-FFA treatment modifies the GUT microbiota.	Piazzi, 2014
A/J Min/+ mice of both sexes	Salmon (as animal muscle) compared with beef or chicken muscles. Feeding from weaning for 10 wks. Sacrifice at 13 wks of age.	Salmon decreased tumour load and size in the SI when compared with beef muscle diet.	High thio-barbituric acid reactive substances (TBARS) formation in salmon and beef ω-6 dietary groups.<br>Reduction of cell viability in *Apc*$^{-/+}$ cells incubated with fecal water from mice fed salmon, pork and RM1 diets.<br>No effect on ACF formation.	Steppler, 2017
BALB/c female mice treated with AOM-DSS to induce colitis and cancer	4.7 % (w/w) of a tuna muscle extract rich in Selenoeine in AIN-93M diet. Dietary treatment from wk 6 until wk 19 (13 wks), when mice were sacrificed.	Reduction in carcinogenesis.	Amelioration of weight loss associated with DSS treatment.<br>Inhibition of the accumulation of myeloid-derived suppressor cells; inhibition of the downregulation of IFGγ production during carcinogenesis.	Masuda, 2018

Table S12. Experiments with fish or its components on different animal models of colorectal cancer metastasis.

Experimental model	Dietary intervention and duration of treatment	Effects on colon cancer and metastasis	Additional parameters measured	Reference
BALB/c ByJ male mice receiving intracolonic or i.v. inoculations with CT-26 cells, a transplantable mouse colon cancer line.	AIN-76 diet containing low and high levels of FO and safflower oil as source of ω-3 PUFA and ω-6 PUFA, respectively 11.6% (w/w) or 46.5% (w/w) of total calories. Diet was fed for 30 days before and after CT-26 transplantation. Mice sacrificed 7, 14, 21, and 28 days after CT-26 inoculation.	Tumor volumes in the intestine at 21 days post-implantation were lower in mice fed FO. Dietary FO suppressed the growth of CT-26 cells and inhibited pulmonary colonization at higher intakes relative to safflower oil.		Cannizzo, 1989
CDF1 male mice s.c. implanted with highly metastatic colon carcinoma cells (Co 26Lu line) maintained in BALB/c mice.	AIN-93 standard diet. EPA and DHA administered by gavage before, during and after cancer cell transplantation.	EPA and DHA inhibit tumour growth at the implantation site and significantly decreased the numbers of lung metastatic nodules. DHA and EPA inhibited lung colonization only when administered together with the tumour cells.	Tumour cells pretreated with fatty acids *in vivo*, in particular with DHA, also showed a low potential for lung colony formation when transferred to new hosts.	Iigo, 1997
CDF1 male mice s.c. implanted with highly metastatic colon carcinoma cells (Co 26Lu line).	AIN-93 standard diet. EPA or DHA plus LA or oleic acid (OA) administered by gavage from day 5 for a total of 3 wks (5 days per wk) after cell implantation.	OA, DHA and EPA demonstrated significant inhibition. In the case of DHA, the inhibition was reduced by co-administration of LA or EPA. Tumor cells treated with DHA showed a very low potential for lung colony formation when injected i.v.	Gelatinolytic activity of MMP-2 and MMP-9; the results suggested that inhibition of metastasis due to treatment with OA and DHA might be due to depressed type-IV collagenase activity.	Suzuki, 1997
F344 male rats injected via mesenteric vein with ACL-15 tumor cells.	Rats at five wks of age were fed AIN-76 standard diet in which fat were supplied by 9.5% (w/w) EPA-ethyl ester plus 0.5% (w/w) LA-ethyl ester (EPA diet), 10% (w/w) LA-ethyl ester (LA diet), and 9.5% (w/w)	Rats fed EPA diet showed a reduction of metastatic foci in liver compared with those fed LA diet. The EPA and PA diet groups had significantly smaller liver metastatic foci than the standard and LA diet groups.		Iwamoto, 1998

	palmitic acid (PA) plus 0.5% (w/w) LA-ethyl ester (PA diet) for 1 wk. At 6 wks of age the animals were inoculated with ACL-15 tumor cells and sacrificed at 9 wks of age.			
Wag-Rij male and female rats injected via portal vein with CC531rat tumour cells.	Rats at 3 months of age were fed a low fat [5% soybean oil (w/w) or high [20% (w/w)] fat diet (fish or safflower oil) for 3 wks before CC531 inoculation until sacrifice after 1 or 3 wks.	One wk after transplantation, FO diet increased metastasis (number and size) compared with low-fat diet, whereas the safflower oil diet had no effect. Three wks after tumor transplantation, FO diet and the safflower oil diet increased metastasis compared with the low-fat diet.	No effects on immune system.	Griffini, 1998
Wag-Rij male rats, CC531 rat tumour cells injected in the spleen.	Diet enriched with 15% (w/w) ω-3 PUFA (DHA, EPA) or with 15% (w/w) coconut oil 3 days before and 28 days after CC531 injection into the spleen. Sacrifice 28 days after dietary treatment.	Macroscopic examination of tumour growth in different organs showed reduction of growth and in the number of tumour nodules in the ω-3 PUFA group.		Gutt, 2007
BALB/c female mice injected (into the spleen) with MC-26 mouse colorectal cancer cells.	Mice 8 to 11 wks old were fed AIN-93G based diet containing 2.5% (w/w) or 5% (w/w) EPA in the FFA form. Feeding 2 wks before and after cells injection.	EPA-FFA administration caused a reduced MC-26 liver tumor burden compared with controls.		Hawcroft, 2012
